# Engineering Lipid–Polymer Nanoparticles for siRNA Delivery to Cancer Cells

**DOI:** 10.3390/ph18060864

**Published:** 2025-06-10

**Authors:** Arthur Manda, Abdulelah Alhazza, Hasan Uludağ, Hamidreza Montazeri Aliabadi

**Affiliations:** 1 Department of Biomedical and Pharmaceutical Sciences, Chapman University School of Pharmacy, Harry and Diane Rinker Health Science Campus, Irvine, CA 92618, USA; 2 Department of Pharmaceutics, Faculty of Pharmacy, Northern Border University, Rafha 76313, Saudi Arabia; 3 Department of Chemical and Materials Engineering, Faculty of Engineering, University of Alberta, Edmonton, AL T6G 2H1, Canada; 4 Faculty of Pharmacy and Pharmaceutical Sciences, University of Alberta, Edmonton, AL T6G 2H7, Canada

**Keywords:** small interfering RNA, cancer, nanoparticles, polymers, lipids

## Abstract

**Background**: RNA interference (RNAi) is a powerful tool that can target many proteins without the expensive and time-consuming drug development studies. However, due to the challenges in delivering RNA molecules, the potential impact of RNAi approaches is yet to be fully realized in clinical settings. Lipid nanoparticles (LNPs) have been the most successful delivery system for nucleic acids, but targeted delivery to a solid tumor still eludes the developed LNPs. We hypothesized that specially designed low-molecular-weight PEIs can partially or completely replace the ionizable lipids for more accommodating vehicles due to the structural flexibility offered by polymers, which could lead to safer and more efficient nucleic acid delivery. **Methods**: To achieve this, we first optimized the LNP formulations as a point of reference for three outcomes: cellular uptake, cytotoxicity, and silencing efficiency. Using a response surface methodology (Design Expert), we optimized siRNA delivery by varying mole fractions of lipid components. Leveraging the optimal LNP formulation, we integrated specifically designed cationic polymers as partial or complete replacements for the ionizable lipid. This methodological approach, incorporating optimal combined designs and response surface methodologies, refined the LPNPs to an optimal efficiency. **Results**: Our data revealed that DOPE and Dlin-MC3-DMA contributed to higher efficiency in selected breast cancer cells over DSPC and ALC-0315 as neutral and ionizable lipids, respectively, based on the software analysis and direct comparative experiments. Incorporation of selected polymers enhanced the cellular internalization significantly, which in some formulations resulted in higher efficiency. **Conclusions**: These findings offer a framework for the rational design of LPNPs, that could enhance the passive targeting and silencing efficiency in cancer treatment and broader applications for RNAi-based strategies.

## 1. Introduction

RNA interference (RNAi) is a potentially powerful tool that can specifically target the expression of many proteins without the expensive and time-consuming drug development studies. Nucleic acids, including small interfering RNA or siRNA, can address some of the challenges associated with current cancer treatment strategies; however, delivery of these molecules to the cancer cells has been a major hurdle. The advent of nanotechnology offers promising avenues for overcoming these hurdles and potentially add siRNA therapy to current cancer treatments [1]. Lipid nanoparticles (LNPs) have been the leading approach to delivering nucleic acids, which enabled FDA-approved patisiran (Onpattro^®^) and givosiran (Givlaari^®^, both by Alnylam Pharmaceuticals) and COVID-19 vaccines manufactured by Pfizer-BioNTech and Moderna. Unfortunately, most of the recent achievements in siRNA delivery in vivo preclinical models have been reported to address disorders originating in the liver [2], since LNPs naturally get deposited in the liver. These include the siRNA-GalNAc conjugates that target the liver [3,4], and both FDA-approved drugs, which both address a hereditary disorder affecting the liver. Also, LNPs face challenges including limited control over release rate and stability.

Breast and prostate cancers rank among the most prevalent and challenging malignancies globally due to their complex pathophysiology and the limitations of existing therapeutic strategies [5,6]. Despite notable advances in targeted therapies and personalized medicine, the treatment of these cancers frequently relies on systemic chemotherapy, which often results in nonspecific toxicity and adverse side effects [7]. Furthermore, resistance to chemotherapy continues to be a formidable challenge, underscoring the urgent need for more effective and selective treatment modalities [8,9]. On the other hand, trastuzumab entered clinical trials as a humanized HER2 antibody, and was approved by the FDA before the turn of the century, which introduced the first molecularly targeted drug to breast cancer treatment and promised a new era [10,11]. However, intrinsic and acquired resistance seems to be inevitable for molecularly targeted drugs as well.

Breast cancer is the most common cancer among women and exhibits a diverse array of subtypes, each characterized by unique genetic and phenotypic profiles [12]. Despite the introduction of many molecules targeting HER family of receptors, CDK4 and 6, PDL1, poly (ADP-ribose) polymerase (PARP), and PI3K/AKT pathway in breast cancer therapy [13], chemotherapy remains the first line of treatment in breast cancer treatment. 

We have previously reported on the potential of specifically designed peptides for replacing the ionizable lipid component of the LNPs for the delivery of siRNA [14] and clustered regularly interspaced short palindromic repeats (CRISPR) and CRISPR-associated protein 9 (Cas9) [15]. In this manuscript, however, we have taken a systematic approach to designing a novel composition for lipid–polymer nanoparticles (LPNPs), which uniquely combine the biocompatibility and safety of lipids with the structural integrity and versatility of specifically designed low-molecular-weight polymers. While polymers have been used in LNP formulations before, either in the form of an outer coating or corona for stealth properties (usually Polyethylene Glycol or PEG [16,17]) or as a core in a core–shell structure where lipid components formed the shell [16,18,19], our approach is different since we have designed the polymers to be truly incorporated into the nanoparticle structure as a component interacting with lipid building blocks of the delivery system. Our hypothesis is that this platform provides a promising opportunity for the delivery of siRNA (and likely other nucleic acids), which could be a therapeutic modality for silencing proteins involved in cancer progression and/or drug resistance. 

While ionizable lipids have been improved over the last decade and became a consistent component of LNPs, they lack the structural flexibility that polymers, in general, offer in terms of multivalent binding. We hypothesize that incorporating specifically designed PEI polymers can offer opportunities for tailoring the structure of these nanoparticles for more efficient delivery. By designing LPNPs specifically for siRNA delivery to breast cancer cells, this research aims to achieve precise protein silencing with minimal off-target effects, thereby tackling the critical challenges of specificity, resistance, and toxicity [20]. 

## 2. Results and Discussion

### 2.1. Design for Optimum LNP Composition

Design Expert has been used in LNP design in several studies before (but not for siRNA delivery, to the best of our knowledge). Different Critical Quality Attributes (CQAs) could be selected to evaluate the Critical Process Parameters (CPPs). Some studies choose to evaluate physical characteristics of the nanoparticles, including particle size, polydispersity index, or zeta potential [21,22,23,24]. While these characteristics are obviously important for nanoparticle performance and could decide the efficiency of LNPs, we decided to evaluate more direct indicators of the efficacy in vitro. We selected to evaluate cellular internalization as the first requirement for efficiency of nucleic acid delivery and toxicity as an indication of the safety profile of the delivery system. However, while internalization into the target cells is required, it does not guarantee the efficacy of silencing, as the siRNA copies could be trapped in the endosomes, not released from the delivery system in a timely manner, and/or degraded before interacting with the target mRNAs. Therefore, for the breast cancer cells, we added the silencing efficiency by evaluating the expression of GFP in transfected MDA-MB-231 cells. We did not have access to a similar counterpart in prostate cancer cells, and therefore, our evaluated outcomes were limited to internalization and toxicity in these cells. 

The 29 runs designed by Design Expert (Supplementary Table S1) based on the defined CPPs were formed and analyzed for the CQA (cellular internalization and cytotoxicity in MDA-MB-231 and silencing efficiency in MDA-231-GFP). Among the 29 runs, the combinations of the selected phospholipid I and ionizable lipids were 8 runs with DSPC and ALC-0315, 7 runs with DSPC and Dlin-MC3-DMA, 7 runs with DOPE and ALC-0315, and 7 runs with DOPE and Dlin-MC3-DMA, which indicates a balanced distribution among the possible combinations.

### 2.2. Cellular Internalization of siRNA Formulations

**Figure 1:**
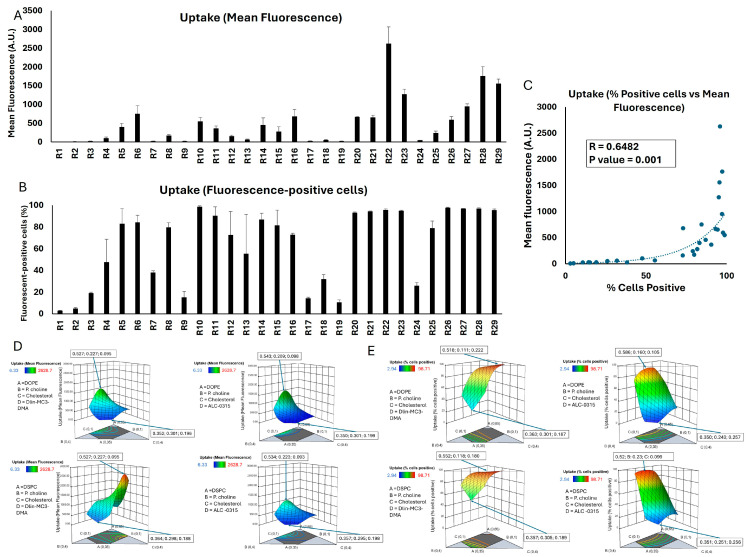
The cellular internalization in MDA-MB-231 cells as mean fluorescence in the cell population (**A**) and the percentage of cells positive for the siRNA fluorescent label (**B**). Bars and error bars in bar graphs represent the average value (n = 3) and the standard deviation, respectively. These two indicators had a significant correlation ((**C**); *p* value = 0.001). The 3D response surface plots for cellular uptake as mean fluorescence and % of fluorescence-positive cells are provided in (**D**,**E**), respectively. R1, R2, …, R29 indicate the “runs” included in the study by Design Expert. Please see Supplementary Table S1 for details.

We quantified cell uptake by flow cytometry using two different indicators, each of which would provide important information: average mean fluorescence in the cell population, and percentage of the cells that were deemed to be fluorescent (siRNA) “positive”, considering the defined gates. While the percentage of cells positive for a fluorescent signal reveals how widely the delivery system has accessed the cells in the population, the mean fluorescence provides a measure of the total amount of siRNA delivered to the population. The mean fluorescence is especially helpful for the runs that are more efficient and would not be differentiated once the percentage of siRNA-positive cells reaches saturation (i.e., 100%). We decided to use both sets of data as CQAs for better conclusions, as they were also significantly correlated (correlation factor (R) = 0.65; *p* value = 0.001; Figure 1C). Figure 1 summarizes the results of the cellular internalization experiments for the 29 runs in breast cancer cells. While some runs did not create a significant mean fluorescent signal (no/little internalization), others showed significant internalization efficiency. Cells exposed to run 22 showed the highest mean fluorescence (~2630 A.U.), while runs 28 and 29 showed the second and third highest mean fluorescence in MDA-MB-231 cells (1767 and 1560 A.U., respectively; Figure 1A). On the other hand, most of the runs showed a significant percentage of cells positive for the siRNA fluorescence signal (>90%), except for a few runs with a low percentage of fluorescence-positive cells (<20%; Figure 1B). The 3D response surface plots represent the trend in changes in the two evaluated sets of data (Figure 1D,E) based on three variables: mole fraction of phospholipid I (DOPE or DSPCE), phosphatidylcholine, and cholesterol (A, B, and C). The mole fraction of ionizable lipid (Dlin-MC3-DMA or ALC-0315) was kept constant at 0.15 for the sake of comparisons. The 3D plots clearly indicate a more significant impact of the phospholipid I mole fraction on the cellular internalization, since an increase in mole fraction of DOPE or DSPC from ~0.35 to > 0.5 increased both mean fluorescent and percentage of fluorescent-positive cells in all possible combinations, while the mole fraction of cholesterol and phosphatidylcholine did not vary significantly among the runs with the highest and lowest internalization. A lower mole fraction for phosphatidylcholine indicated a better internalization in all four combinations; however, a consistent trend was not observed for the cholesterol. 

We performed an analysis to investigate any potential correlations between the mole fraction of the LNP components and the two indicators of the cellular internalization, which are summarized in Supplementary Figure S1. There was no significant correlation between the mole fraction of any of the four LNP components and mean fluorescence or percentage of the siRNA-positive cells. This was expected, because the variation among the runs is not limited to one factor, and therefore, a simple correlation study might not reveal the effect of the mole fraction of each component. A comparison between the cellular internalization and the runs incorporating DOPE vs. the runs with DSPC did not reveal a statistical difference either, which is perhaps due to the large variability in the data sets (Supplementary Figure S1I,J). Interestingly, exposing the MDA-MB-231 cells to Dlin-MC3-DMA containing runs showed a significantly higher percentage of siRNA-positive cells compared to ALC-0315 containing runs (82% vs. 47.6% average, respectively; *p*-value < 0.005). A similar trend was also observed in the mean fluorescence of the two data sets (739 vs. 284 A.U.); however, the difference barely missed the significance threshold (*p* value = 0.053). 

These findings were particularly interesting since they go against the observations in two recent reports. An in vitro study on delivering self-amplifying RNA (saRNA) and mRNA showed a better performance of ALC-0315 over the Dlin-MC3-DMA in terms of maximizing protein expression [25]. Also, an in vivo study with C57BL/6J mice aiming to compare siRNA delivery by LNPs containing the two ionizable lipids reported that LNPs incorporating ALC-0315 silenced coagulation factor II and ADAMTS13 2- and 10-fold more than the LNPs with Dlin-MC3-DMA [26]. However, this study also reported a higher hepatotoxicity (observed as an increase in ALT) for the LNPs that contained ALC-0315, possibly indicating higher uptake.

The statistical analysis performed by Design Expert highlighted that the model effectively captured significant interactions influencing the percentage of the siRNA-positive cells, with good explanatory power as indicated by the adjusted and predicted R^2^ values. However, in analyzing the mean fluorescence data, the software reported a negative value for coefficient for DE (ionizable lipid and the choice of the phospholipid I). Despite this limitation to the explanatory power of the model, it still captured significant relationships and was a statistically significant model (*p* = 0.0017; Supplementary Table S2 for a summary of analysis).

### 2.3. Cytotoxicity Assessment of siRNA Formulations

Toxicity is one of the limiting factors for nucleic acid delivery systems. For example, high-molecular-weight (i.e., 20,000 Daltons) PEI was once considered gold standard in this regard; however, toxicity of these polymers has marred their considerable efficacy [27]. In fact, a reverse relationship between the efficacy and toxicity among the PEIs with varying molecular weights is known. To include the toxicity into the CPPs, and considering the multicomponent nature of the nanoparticles, we decided to plot the cell viability of different study groups against the final amount of siRNA exposed to MDA-MB-231 cells (in nanomoles), which was calculated based on the volume of LNP dispersion added to the wells and the concentration of siRNA in LNP dispersion. With this approach, the concentration of all the components forming the LNPs were varied among the study groups with the same proportion. 

**Figure 2:**
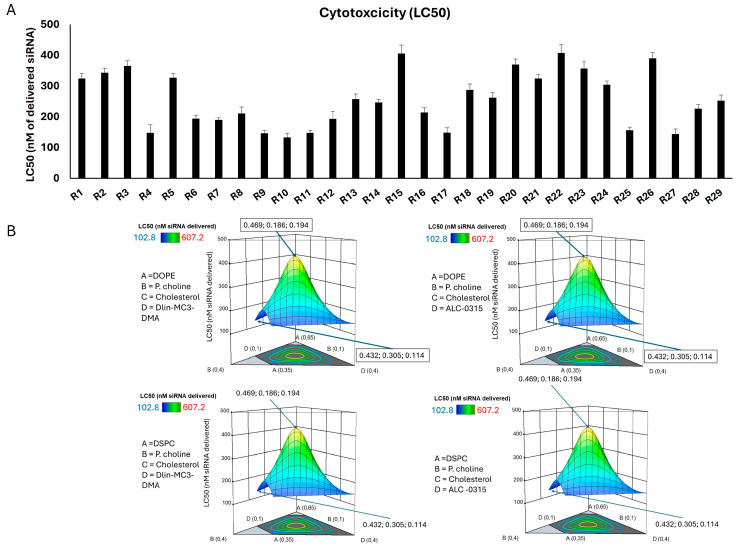
The toxicity of the designed runs in MDA-MB-231 cells as Lethal concentration for 50% cell death based on the siRNA delivered (nM) (**A**). Bars and error bars in bar graph represent the average value (n = 3) and the standard deviation, respectively. The 3D response surface plots for LC50 are provided in (**B**). R1, R2, …, R29 indicate the “runs” included in the study by Design Expert. Please see Supplementary Table S1 for details.

Figure 2 summarizes the LC50 of the 29 runs in the MDA-MB-231 cells and the 3D response surface plots. LC50 calculated for none of the runs was less than 100 nM siRNA delivered, which was the concentration delivered for silencing efficiency experiments. Run 10 showed the most toxicity (LC50 ≈ 133 nM siRNA delivered) and Runs 15 and 22 were the least toxic runs in MDA-MB-231 cells (with LC50s of 407 and 405 nM siRNA delivered, respectively). The LC50 for most of the runs was in the 150–400 nM siRNA range. The 3D response surface plots represent the trend in changes in the LC50 based on the same three variables: mole fraction of phospholipid I (DOPE or DSPCE), phosphatidylcholine, and cholesterol (A, B, and C). The mole fraction of ionizable lipid (Dlin-MC3-DMA or ALC-0315) was again kept constant at 0.15 for consistency. However, the 3D response surface plots for LC50 results were identical for all possible combinations of the selected phospholipid I and ionizable lipids. All the plots showed a higher LC50 (lowest toxicity) in the middle of the bell-shaped 3D plot, indicating that the toxicity would increase when either of the variables (phospholipid I and ionizable lipid fraction) was used in the higher end of the mole fraction range. 

We investigated any potential correlation between the individual CPPs and toxicity observed in the MDA-MB-231 cells, and a direct comparison between the LC50s calculated for LNPs containing DOPE vs. DSPC and ALC-0315 vs. Dlin-MC3-DMA, and the results are summarized in Supplementary Figure S2. No significant correlation was found between any of the four mole fractions and the calculated LC50s. We also carried out direct comparisons between the runs containing DOPE vs. DSPC as the phospholipid I and the runs containing ALC-0315 vs. Dlin-MC3-DMA, and the difference in the average of the LC50s was not statistically significant (Supplementary Figure S2E,F). Hence, the nature of the lipid did not seem to significantly affect the cytotoxicity observed with the lipid formulations, given the experimental limitations of the investigated system.

The statistical analysis performed by Design Expert showed that the selected factors significantly influenced cytotoxicity. However, the linear mixture terms did not contribute significantly (*p* = 0.6151), suggesting that individual mixture components alone may not sufficiently explain the variation in LC50. The interaction effects also indicated a potential combined effect of the LNP components on the cytotoxicity. The lack of a fit test was not significant, suggesting that the model adequately captured the data variability without significant deviation. The adjusted and predicted R^2^ values were lower than the outcome for the cellular internalization. The elevated variance inflation factors (VIFs) might be an indication for multicollinearity issues due to high correlation between independent variables, which makes it difficult for the model to distinguish the unique contributions of the variables, leading to unstable coefficient estimates. However, the residuals vs. predicted plot showed acceptable random dispersion of residuals, supporting the adequacy of the model fit. Overall, the study findings highlighted that although the model effectively captured certain significant interactions influencing LNP cytotoxicity, the limited explanatory power, as indicated by the predicted R^2^ value, pointed to areas needing further refinement. Please see the summary of statistical analysis in Supplementary Table S2.

### 2.4. Silencing Efficiency of LNP Formulations

**Figure 3:**
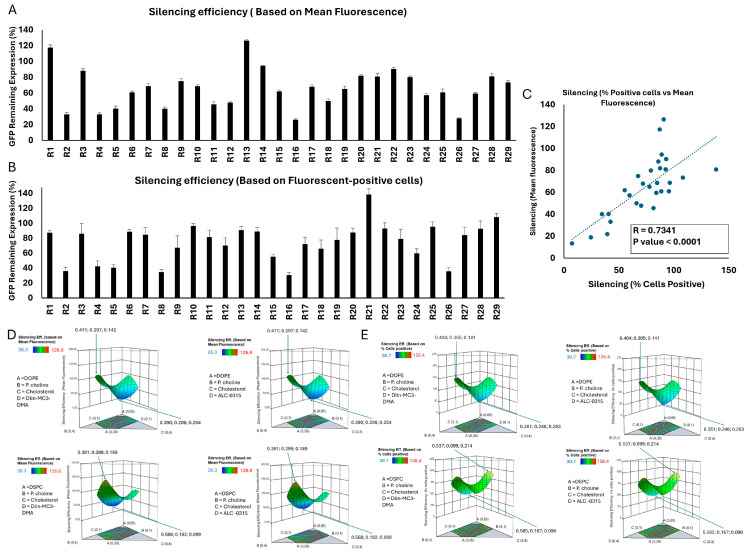
The silencing efficiency for GFP in MDA-231-GFP cells as calculated based on mean fluorescence in the cell population (**A**) and the percentage of cells positive for the GFP fluorescent signal (**B**). Bars and error bars represent the average value (n = 3) and standard deviation, respectively, for the GFP remaining expression (%). The mean fluorescence and percentage of fluorescence-positive cells for the two groups with error bars are provided in Supplementary Figure S3. These two indicators had a significant correlation ((**C**); *p* value < 0.0001). The 3D response surface plots for the GFP remaining expression (%) calculated based on mean fluorescence and % of fluorescence-positive cells are provided in (**D**,**E**), respectively. R1, R2, …, R29 indicate the “runs” included in the study by Design Expert. Please see the Supplementary Table S1 for details.

The final selected CQA was the silencing efficiency that was evaluated in MDA-MB-231 cells permanently expressing GFP (MDA-231-GFP). Cells were exposed to scrambled siRNA and GFP-targeting siRNA for a side-by-side comparison using flow cytometry and to calculate the silencing efficiency. Again, we used both mean GFP fluorescence and percentage of GFP-positive cells as indicators of the GFP expression levels to calculate the silencing efficiency. There was a significant correlation between the silencing efficiencies calculated based on mean GFP fluorescence and percentage of GFP-positive cells (correlation factor (R) = 0.7341, *p* value < 0.0001; Figure 3C) and both sets of data were included as CQAs. The silencing efficiency data are summarized in Figure 3. The mean GFP fluorescence and percentage of GFP-positive cells for the two groups (cells exposed to scrambled siRNA or GFP-targeting siRNA) are provided in Supplementary Figure S3. 

Figure 3A summarizes the silencing efficiency of the 29 runs calculated based on the average of GFP fluorescence of the cell populations. The mean fluorescence of untreated cells and the percentage of GFP-positive cells were 14,034 AU and 85.2% on average, respectively. Some formulations showed no silencing efficiency (runs 1 and 13 when silencing efficiency was calculated based on mean GFP fluorescence; or runs 10, 21, and 29 when it was calculated based on the percentage of GFP-positive cells), with others demonstrating a slight efficiency. Run 16 showed the highest efficiency in the GFP remaining expression (26%) based on mean GFP fluorescence, followed by runs 26 and 2 (28% and 33%, respectively). Based on the percentage of GFP-positive cells (Figure 3B), run 16 was again the most efficient run (~30.7%). However, in both sets of data, there were some runs that showed a decrease in GFP fluorescence by delivering scrambled siRNA (both in terms of average GFP signal and the percentage of GFP-positive cells; Supplementary Figure S3); this could be due to non-specific effects because of a high concentration of delivered siRNA copies, or toxicity that might affect the overall fluorescence signal. Among those runs that showed a decrease in fluorescence by delivering scrambled siRNA, run 2 and run 8 were noticeable, since they also showed significant silencing efficiency. 

Interestingly, there was no significant correlation between the cellular internalization results and the silencing efficiency (Supplementary Figure S4). Although reports of such correlation between internalization and silencing can be found in the literature [28], and internalization into the cytoplasm is a requirement for the siRNA to be effective, cellular internalization does not necessarily guarantee an effective silencing. For example, it is well known that siRNA nanoparticles internalized efficiently via endocytosis pathways could be trapped in the endosome and never release the siRNA in cytoplasm intact [29]. Another important step for the efficiency of internalized siRNA copies is the timely release from the nanoparticles, which needs to create a consistent number of siRNA copies in cytoplasm that could effectively silence the targeted protein [30]. On the other hand, achieving effective silencing is also possible with fewer copies of siRNA that were delivered intact and in a timely manner to the site of action. We have previously reported a correlation between siRNA internalization and silencing efficiency against Janus Kinase 2 (JAK2) in MDA-MB-231 cells and Signal Transducer and Activator of Transcription 3 (STAT3) proteins in MDA-MB-468 cells (another triple negative breast cancer cell line) in vitro with hydrophobically modified low-molecular-weight PEIs that showed minimal cellular internalization [31]. In this study, runs 16 and 26, which showed effective silencing without a significant reduction in GFP levels by delivering scrambled siRNA, showed moderate mean fluorescence in the internalization data set (682 and 596 AU in mean fluorescence, and 73% and 97% in siRNA-positive cells, respectively).

We performed a correlation analysis to investigate any potential correlations between the mole fraction of the LNP components and two indicators of the silencing efficiency (Supplementary Figure S5). As in cellular internalization and toxicity, a simple one-to-one correlation between this CQA and the LNP components did not reveal any significant correlation. A direct comparison between the runs containing DOPE vs. ones with DSPC did not show a significant difference either, despite a slightly higher silencing efficiency among the LNPs incorporating DOPE as phospholipid I (76.2% vs. 66.2% calculated based on percentage of GFP-positive cells, and 66.8% vs. 62.4% calculated based on mean GFP-fluorescence). Despite an advantage for LNPs incorporating Dlin-MC3-DMA over the runs with ALC-0315 for the GFP remaining expression (78.8% vs. 63.8% based on percentage of GFP-positive cells and 68.0% vs. 59.3% based on mean GFP-fluorescence), this difference did not also reach significance (*p* value = 0.169 and 0.347, respectively).

The ANOVA test showed that the selected model for silencing efficiency calculated based on percentage of GFP-positive cells was significant and the lack of fit test was not significant (indicating that the model was fit for the analysis); however, the linear mixture component did not have a significant contribution, which indicates that individual components alone were not sufficient to explain the variability in silencing efficiency. Overall, the statistical analysis of this data set indicated that although the model effectively captured certain significant interactions influencing GFP silencing efficiency, the limited explanatory power, as indicated by the negative predicted R^2^ value, pointed to areas needing further refinement. On the other hand, the ANOVA test for silencing efficiency calculated based on mean GFP-fluorescence missed the significance mark by a narrow margin (*p* = 0.0696), while the lack of fit was not significant, which indicates that the model adequately captured the data variability without significant deviation. The analysis of coefficient estimates showed that individual components such as phospholipid I, phosphatidylcholine, and ionizable lipid had significant positive impacts on silencing efficiency calculated based on mean fluorescence. Overall, the study of the silencing efficiency calculated based on mean fluorescence showed similar deficiencies to the model as was observed for the calculation of silencing efficiency based on percentage of the GFP-positive cells (including the negative predicted R^2^ value). Please see the summary of statistical analysis in Supplementary Table S2.

For the optimum formulation, based on the presented data, Design Expert concluded that the best performing formulation was DOPE as phospholipid I, Dlin-MC3-DMA as ionizable lipid, and DOPE (0.4)/Dlin-MC3-DMA (0.4)/phosphatidylcholine (0.1)/cholesterol (0.1) as the composition. This proposed formulation was a surprising duo to preference for DOPE over DSPC. As mentioned before, DSPC has been a consistent component of FDA-approved LNPs, including both COVID-19 vaccines [32]. Also, run 16 and run 26, which showed the most significant silencing efficiency among the proposed runs, evaluated both incorporated DSPCs as phospholipid I (Supplementary Table S1). Therefore, we decided to perform a side-by-side comparison between the proposed “optimum formulation” (OF) and runs 16 and 26 (as the second-best performing formulation in silencing efficiency studies). To further compare DOPE and DSPC and also ALC-0315 and Dlin-MC3-DMA, we also included those variations in this study. This design allowed us to compare each formulation with the exact duplicate but the alternative lipid (e.g., the optimum formulation or OF is compared with OF/DSPC, which is the same composition where DOPE replaces DSPC). Table 1 summarizes the compositions of the LNPs included in this study.

**Figure 4:**
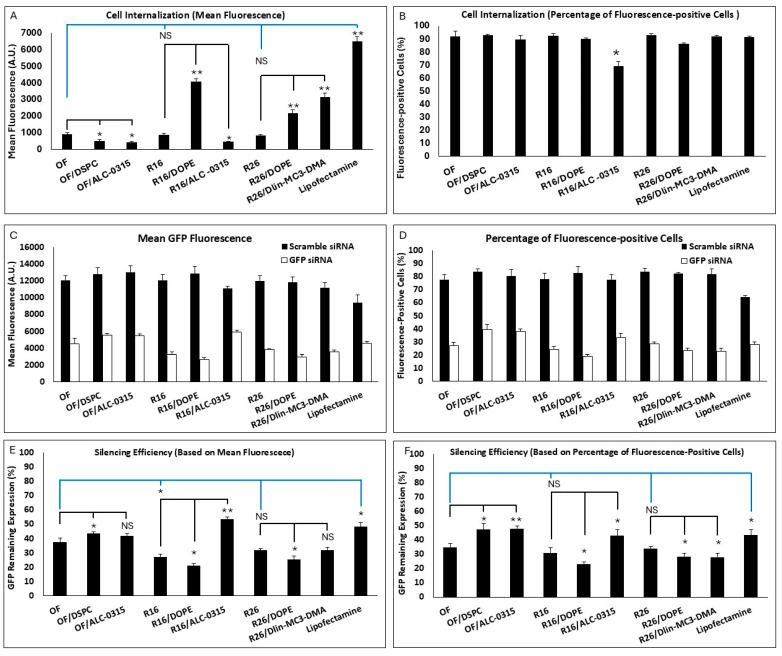
Validation of the optimum formulation (OF) and the most efficient runs in terms of GFP silencing (R16 and R26) in comparison to the variations in the composition. For each formulation, two “alternative” formulations were created by replacing DOPE with DSPC (or vice versa) or replacing ALC-0315 with Dlin-M3-DMA (or vice versa). Lipofectamine was used as a point of reference to put the data into perspective. Bars and error bars indicate the average and standard deviation of the results for each group, respectively (n = 3). NS, *, and ** represent “not significant” (*p* > 0.05), *p* < 0.05, and *p* < 0.005, respectively. Cellular internalization in MDA-MB-231 cells was evaluated by mean fluorescence of cell populations (**A**) and percentage of GFP-positive cells (**B**). The level of mean fluorescence of MDA-231-GFP cells exposed to scrambled or GFP-targeting siRNA is presented in terms of mean fluorescence (**C**) and percentage of GFP-positive cells (**D**), and the GFP remaining expression (%) was calculated based on mean fluorescence (**E**) and percentage of GFP-positive cells (**F**). The statistical analysis data presented with blue lines indicate the difference with the OF results.

All nine formulations included in the study were investigated for cell internalization and silencing efficiency as before, and the results are summarized in Figure 4. There was no significant difference between the OF, R16, and R26 in cell internalization (based on both mean fluorescence or percentage of siRNA-positive cells). Based on mean fluorescence (Figure 4A), replacing DOPE with DSPC or replacing Dlin-MC3-DMA with ALC-0315 in the OF composition both reduced the internalization significantly. Interestingly, replacing DSPC with DOPE in R16 and R26 also increased the cellular internalization significantly. Replacing Dlin-MC3-DMA with ALC-0315 in R16 reduced the mean uptake, and replacing ALC-0315 with Dlin-MC3-DMA in R26 enhanced the mean uptake in MDA-MB-231 cells. The commercial Lipofectamine created the highest mean fluorescence among the study groups. Based on siRNA-positive cell data, all included LNPs created comparable results (~85–90%), except the lower uptake in R16 “alternative”, where Dlin-MC3-DMA was replaced with ALC-0315 (Figure 4B). All these data confirm the superiority of DOPE over DSPC and Dlin-MC3-DMA over ALC-0315 in internalizing siRNA in the selected triple-negative breast cancer cell line.

**Figure 5:**
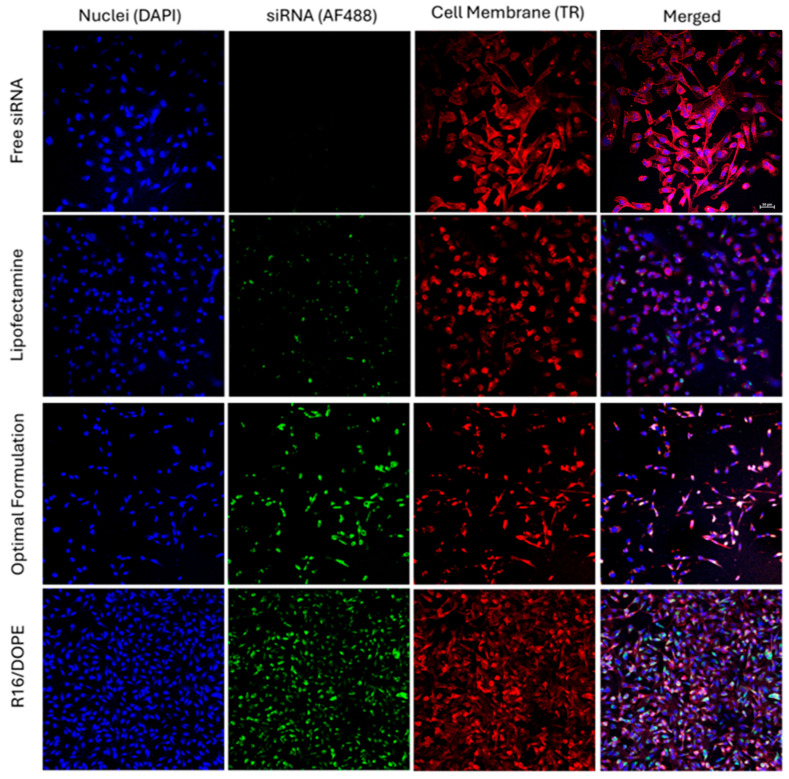
The internalization of Alexa Fluor 488-labeled siRNA in MDA-MB-231 cells by exposing the cells to free siRNA, Lipofectamine^TM^, optimum formulation recommended by Design Expert, and R16/DOPE. Blue, green, and red channels represent DAPI-stained nuclei, siRNA, and Texas-Red (TR)-stained cell membrane. Scale bar is 50 µm.

**Figure 6:**
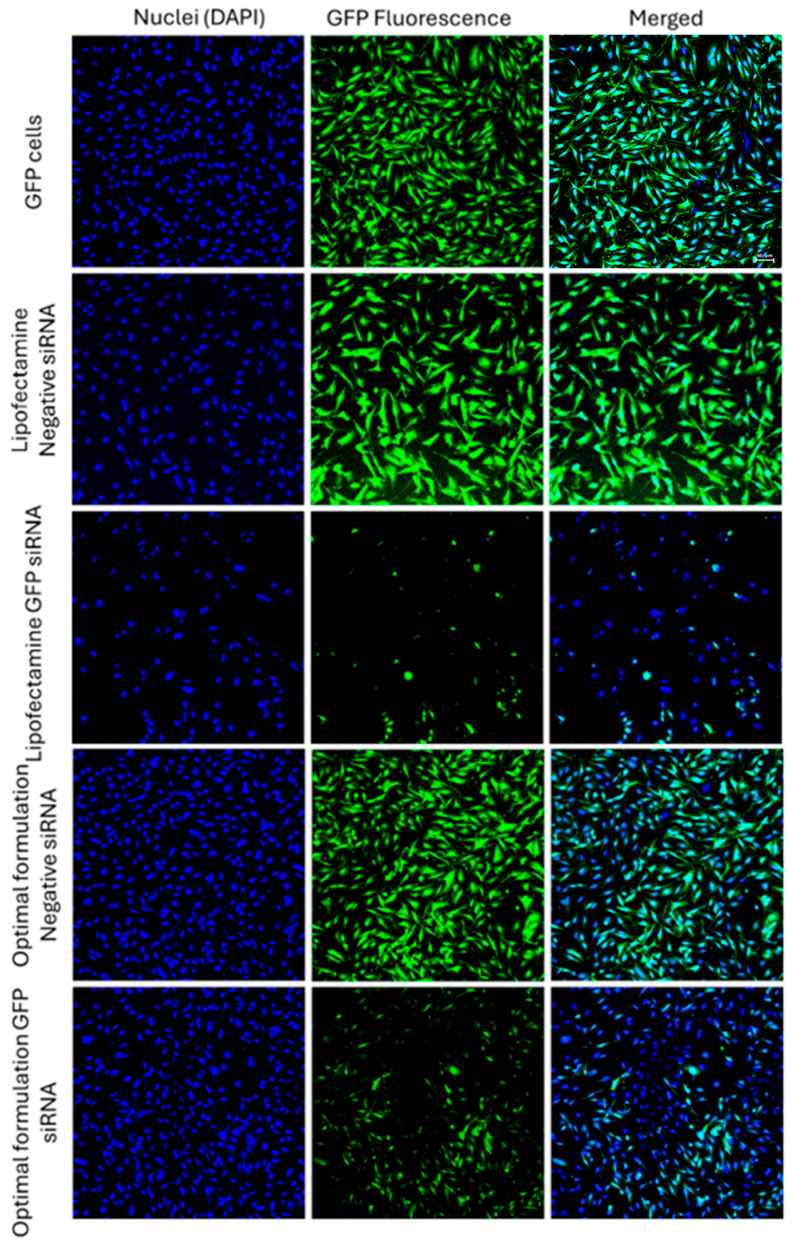
Silencing of Green Fluorescence Protein (GFP) in MDA-231-GFP cells by GFP-targeting siRNA delivered by Lipofectamine^TM^, optimum formulation, and R16/DOPE compared to cells exposed to negative (scrambled siRNA). Blue and green channels represent DAPI-stained nuclei and GFP, respectively. The scale bar is 50 µm.

The silencing efficiency for these LNPs is summarized in Figure 4C,D for cells exposed to scrambled or GFP-targeting siRNA. The effect of scrambled siRNA on GFP-fluorescence was not significant in these study groups, which makes the results more reliable. Silencing efficiencies calculated based on mean GFP-fluorescence are presented in Figure 4E. R16 showed the highest efficiency in silencing GFP in MDA-MB-231-GFP cells compared to the OF composition; however, while R26 was also more efficient than OF in this regard (32% vs. 37.5% remaining expression), the difference was not statistically significant. Lipofectamine was less effective in silencing GFP based on the mean fluorescence results. Replacing DOPE with DSPC in OF composition reduced the silencing efficiency significantly; however, while replacing Dlin-MC3-DMA with ALC-0315 in OF did reduce the silencing efficiency (42% remaining GFP expression for ALC-0315 compared to 37.5% for OF), the difference was not statistically significant. In R16 and R26 formulations, replacing DSPC with DOPE increased the silencing efficiency significantly. Replacing Dlin-MC3-DMA with ALC-0315 in R16 reduced the silencing efficiency; however, replacing ALC-0315 with Dlin-MC3-DMA in R26 composition did not affect the silencing efficiency significantly. A similar silencing pattern was also observed based on the percentage of GFP-positive cells’ data (Figure 4F). In this data set, however, the superiority of R16 and R26 over OF in silencing efficiency was not statistically significant. The internalization and silencing efficiency of Lipofectamine, optimum formulation, and R16/DOPE were also visualized by confocal microscopy and the images are summarized in Figure 5 and Figure 6, respectively. 

Based on the presented data, we can conclude that the Design Expert analysis correctly selected DOPE over DSPC and Dlin-MC3-DMA over ALC-0315 in internalizing siRNA in the selected triple negative breast cancer cell line. All the variations in the original compositions also confirmed a higher silencing efficiency for DOPE over DSPS and Dlin-MC3-DMA over ALC-0315. Both findings may seem surprising at first glance: reports generally show a higher efficiency for ALC-0315 compared to Dlin-MC3-DMA in siRNA delivery, and DSPC is a far more popular choice of neutral lipid in commercialized LNPs. However, in both data sets, R26 composition, with DOPE replacing the DSPC (R16/DOPE), showed more efficacious than the recommended composition by the software with the best silencing efficiency (20.9% and 23.1% remaining expression for data calculated based on mean fluorescence and percentage of GFP-positive cells, respectively). This might be an indication that the selected composition using this type of algorithm might not be the best possible option. It is noteworthy that lipid selection could be significantly affected by cell type, as the internalization of any nanoparticle could vary depending on the cell characteristics. Therefore, we selected the R16/DOPE formulation for the design of the LPNP compositions.

### 2.5. Evaluating the LNP Compositions in Prostate Cancer Cells

**Figure 7:**
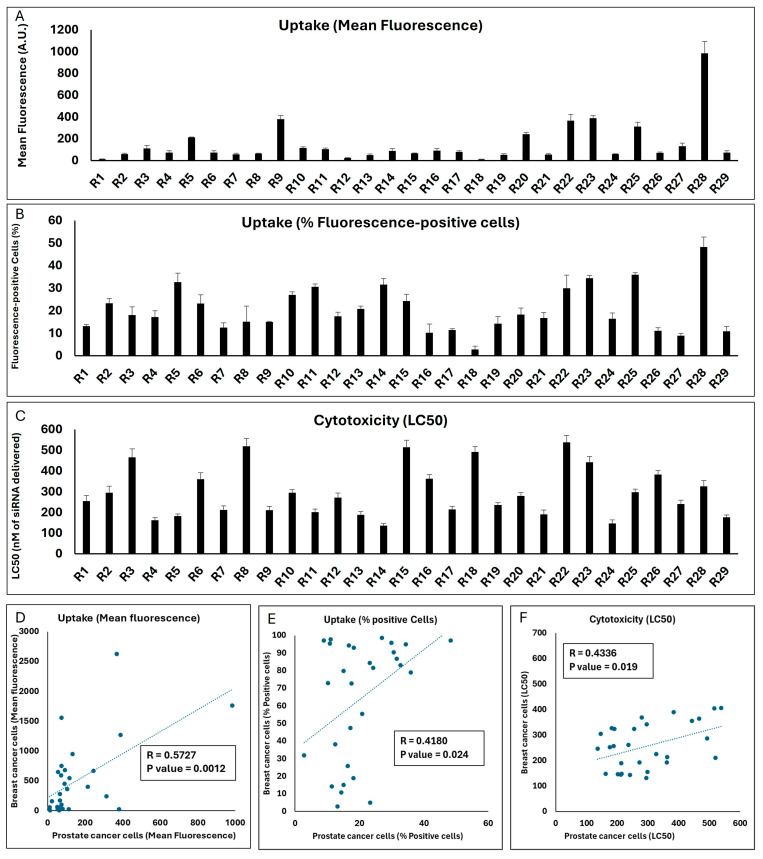
Performance of Design Expert “runs” in human prostate cancer cell line LNCaP: The cellular internalization, quantified both as the mean fluorescence of the cell population (**A**) and percentage of fluorescence-positive cells (**B**), and cytotoxicity of the runs represented as LC50 (**C**) were investigated in the prostate cancer cells. The correlation graphs, as well as R and *p* values, are presented in (**D**–**F**).

The reproducibility of the performance of a carrier in different cell types is an important consideration. While the idea of a carrier that is equally safe and effective in delivering the cargo to different cancer types is certainly attractive, it stands to reason that the formulation would need adjustments depending on the cell type with different membrane compositions, receptor expression, and intracellular processes. To test the performance of the LNPs in a different cell type, we studied the same 29 runs selected by Design Expert in the Androgen-sensitive human prostate cancer cell line LNCaP for cellular internalization and toxicity, and the results are summarized in Figure 7. 

The mean fluorescence of the LNCaP cell populations exposed to the same 29 runs was significantly lower than what was observed in the MDA-MB-231 cells (the highest mean fluorescence was 984 A.U. for Run 28; Figure 7A). However, there was a strong significant correlation between the mean fluorescence created by the runs in the two cell lines (*p* value = 0.0012; Figure 7D). In line with reduced uptake, the highest percentage of siRNA-positive cells did not exceed 50% for any of the runs in LNCaP cells (Figure 7B), while a significant correlation did exist for this outcome between the two cell lines (*p* value = 0.024; Figure 7E). A variety of LC50s was calculated for the 29 runs in the LNCaP cell line, and it was noteworthy that the LC50 for three runs exceeded 500 nM of siRNA delivered. Run 14 showed the highest toxicity among the runs (LC50 = 136 nM of siRNA delivered; Figure 7C). A significant correlation was also observed between the toxicity of the runs in the two selected cell lines (*p* value = 0.019; Figure 7F). 

This set of experiments indicates that, in general, the performance of the LNP formulations included in the Design Expert software (Version 13) runs in terms of cellular internalization and toxicity correlated between the two selected cell models, which might be due to similar factors affecting the uptake and toxicity in these models in vitro. However, this does not necessarily mean that the best performing choice in one cell line would be the best option in the other; for example, the R28 formulation that was the most efficient formulation in internalizing siRNA in the prostate cancer LNCaP cells did not exhibit the highest internalization in the breast cancer MDA-MB-231 cells.

### 2.6. Design for Optimum LPNP Composition 

**Figure 8:**
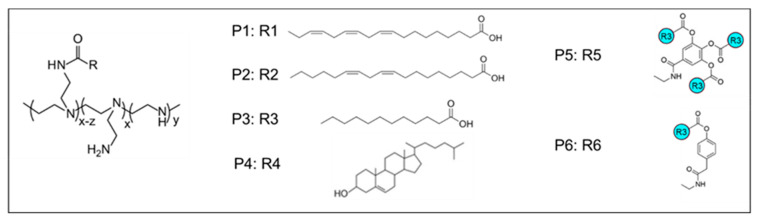
The structures of the six polymers with low-molecular-weight PEI (1.2 kDa, left) as the backbone and different conjugated hydrophobic moieties. The degree of lipid substitution for the polymers was 3.2 lipids per PEI in P1, 2.6 lipids per PEI in P2, 6.1 lipids per PEI in P3, 2.1 lipids per PEI in P4, 3.1 lipids per PEI in P6, and 2.8 lipids per PEI in P6.

As mentioned before, we considered R16/DOPE as the most efficient composition for LNPs to deliver siRNA to the MDA-MB-231 cells and used this combination as the basis for designing the experiments for LPNP evaluations. DOPE was established as phospholipid I with a mole fraction of 0.46, and phosphatidyl choline and cholesterol were used at a mole fraction of 0.1 each in all runs. Therefore, we had two CPPs to be used by Design Expert: the polymer choice (one of the six polymers presented in Figure 8), and the mole fraction of the selected polymer. Lipopolymers have been proven effective in delivering nucleic acids to different cells time and again, and in some cases, have shown superiority to LNPs in direct comparison [33]. As previously mentioned, our objective was to investigate partial or complete replacement of Dlin-MC3-DMA (which was established as the more efficient ionizable lipid for this purpose) by the polymer. Therefore, the range of mole fraction for the selected polymer was set to 0.085–0.34, with the consideration that the sum of mole fractions for Dlin-MC3-DMA and polymer would be 0.34 (the mole fraction of the ionizable lipid in R16/DOPE). The same CQAs (cytotoxicity, siRNA cellular internalization, and gene silencing efficacy) were used and the 23 runs included in the study by Design Expert are presented in Supplementary Table S3. Among them, four runs incorporated polymer 1, five runs polymer 2, five runs polymer 3, and three runs for polymers 4, 5, and 6 each. Also, the mole fraction of polymer was 0.085 in six runs (the lowest proportion for polymer), and 0.34 in six runs (one for each polymer), which were the runs that completely replaced Dlin-MC3-DMA with one of the polymers.

### 2.7. Cellular Internalization of LPNPs in MDA-MB-231 Cells

**Figure 9:**
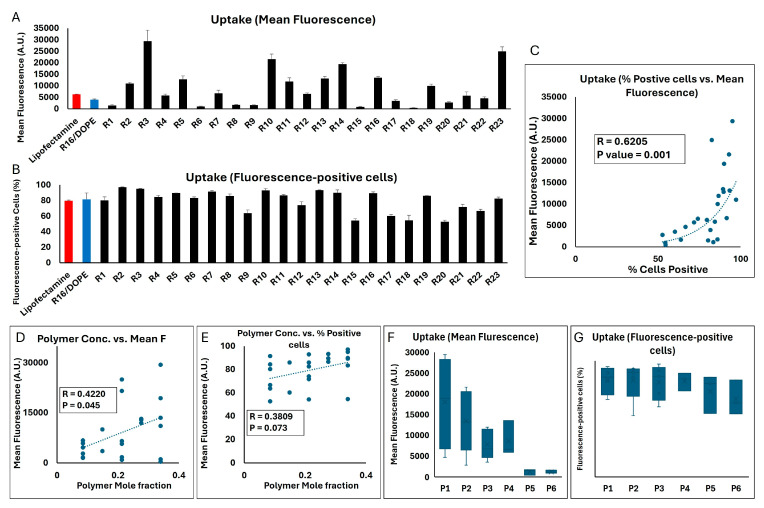
The cellular internalization for lipid–polymer nanoparticles (LPNPs) in MDA-MB-231 cells as mean fluorescence in the cell population (**A**) and the percentage of cells positive for the siRNA fluorescent label (**B**). Bars and error bars in bar graphs represent the average value (n = 3) and the standard deviation, respectively. These two indicators had a significant correlation ((**C**); *p* value = 0.001). A positive correlation was observed between the mole fraction of incorporated polymer and the mean fluorescence (**D**) and % fluorescence-positive cells (**E**), which was only statistically significant for the mean fluorescence. Also, the average of mean fluorescence was the highest for runs incorporating polymer 1 (**F**), and the results showed less variability in terms of percentage of fluorescence-positive cells (**G**). R1, R2, …, R23 indicate the “runs” included in the study by Design Expert. Please see Supplementary Table S3 for details.

The two methods of quantification of the siRNA internalized in MDA-MB-231 cells showed a similarly significant correlation in this set of studies (correlation factor = 0.6205; *p* value = 0.001; Figure 9C). With the mean fluorescence of cell population exposed to AF647-labeled siRNA delivered by Lipofectamine^TM^ and R16/DOPE (as a commercially available reagent and the most effective LNP formulation in our studies) being 6291 and 3937 A.U., respectively, many of the LPNP runs created a significantly higher internalization in the MDA-MB-231 cells (29,400, 24,971, 21,582, and 19,391 A.U. for R3, R23, R10, and R14, respectively, were the four highest results; Figure 9A). A similar significant difference was not, however, observed in the percentage of cells positive for the fluorescence signal, mostly because close to maximum internalization in Lipofectamine^TM^ and R16/DOPE study groups (~ 80 and 82%, respectively). Runs 2,3, 5, 7, 10, 13, 14, and 16 showed ≥ 90% fluorescence-positive cells. There were also runs that did not show a similar internalization efficiency and runs 15 and 20 created the lowest % fluorescence-positive cells (54 and 52.6%, respectively; Figure 9B). A significant positive correlation was observed between the mole fraction of the polymer in the formulation and the mean fluorescence (R = 0.422; *p* value = 0.045; Figure 9D). A similar trend was seen in terms of % fluorescence-positive cells (R = 0.3809); however, the correlation was not statistically significant (*p* value = 0.073; Figure 9E). A direct comparison between the six polymers included in the study showed that the average of mean fluorescence in the MDA-MB-231 cell population exposed to the runs incorporating polymer 1 was the highest among the different polymers (17,964 A.U.; Figure 9F). A one-way ANOVA test showed a significant difference among the six polymers included (*p* value = 0.0165), and a Tukey post hoc test revealed a significant difference only between polymer 1 vs. polymers 5 and polymer 1 vs. polymer 6 (*p* values of 0.324 and 0.323, respectively). A similar comparison between the average of percentage of fluorescence-positive cells among the runs incorporating each of the six polymers showed less variability among the runs, where the average percentage was around 82% for runs incorporating polymers 1–4, and 73.6% and 67% for runs including polymers 5 or 6, respectively (Figure 9G). 

Since this set of experiments only included two CPPs (mole fraction and the structure of the selected polymer), the response surface plots were either based on one factor (the selected polymer) for a pre-determined polymer mole fraction (0.085, 0.2125, or 0.34) or a two-component mix (six separate plots for different polymers based on the mole fractions), and the one factor respond surface plots for cellular internalization are summarized in Supplementary Figure S6. It is noteworthy that except for polymer 5, the highest mean fluorescent was achieved when the polymer completely replaced Dlin-MC3-DMA (mole fraction of 0.34 for polymers 1–4 and 6). 

For internalization calculated based on mean fluorescence, the statistical analysis using a reduced quadratic main effects model revealed that the selected combination of formulation factors significantly affected the uptake (ANOVA F-value = 37.02, *p* < 0.0001). Polymer concentration showed particularly strong effects (coefficient = 0.0185, 95% CI: 0.0166 to 0.0204, *p* < 0.05). These findings indicate that increasing the concentrations of polymer can significantly improve nanoparticle performance. The enhancement in uptake may be attributed to facilitating endosomal escape and stabilizing particle structures during internalization. This theory requires experimental confirmation in future studies. The interaction between Dlin and polymer concentration (DE) revealed a significant negative effect (coefficient = −0.0084, 95% CI: −0.0162 to -0.0005, *p* < 0.05). This suggests that while both factors independently enhance uptake, their combined effect may reduce overall efficiency. One potential explanation for this reduction could be the formation of overly stable particles at high concentrations of Dlin and polymer, impeding their disassembly within the cell and subsequent payload release. Analysis of polymer types revealed significant variability in their contributions to uptake. DF [5] exhibited a strong positive effect (coefficient = 0.0076, 95% CI: 0.0032 to 0.0119, *p* < 0.05), suggesting that certain polymers strongly enhance uptake efficiency. Conversely, DF [4] showed a significant negative coefficient (−0.0052, 95% CI: −0.0095 to −0.0008, *p* < 0.05), indicating that some polymer types can inhibit uptake under specific conditions. 

In analysis of the cellular internalization calculated based on percentage of fluorescence-positive cells, the ANOVA results showed statistical significance for the overall model (F-value = 7.18, *p* = 0.0016), indicating that the combined effects of the formulation factors significantly explained the observed variability in uptake. Polymer concentration had a strong positive influence on cellular uptake with a coefficient estimate of 85.27. This highlights that increasing the concentration of polymer enhances nanoparticle uptake. The lack of fit test remained not statistically significant (F-value = 3.98, *p* = 0.0779), indicating that the model adequately fits the data and does not leave systematic variability unexplained. The R^2^ value of 0.9243 indicates that 92.4% of the variability in LPNP uptake was explained by the model, highlighting its strong explanatory power. However, the adjusted R^2^ of 0.7956 reflects moderate generalizability, suggesting room for improvement in capturing additional variability. The adequate precision value of 9.67 confirms a strong signal-to-noise ratio, providing confidence in navigating the design space and ensuring reliable predictions. Please see the summary of statistical analysis in Supplementary Table S4.

### 2.8. Cytotoxicity of LPNPs in MDA-MB-231 Cells

**Figure 10:**
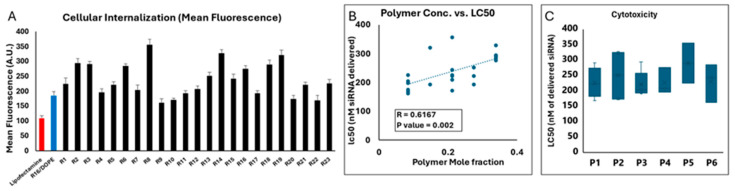
The toxicity of the designed LPNP runs in MDA-MB-231 cells as Lethal concentration for 50% cell death (LC50) based on the siRNA delivered (nM) (**A**). Bars and error bars in bar graph represent the average value (n = 3) and the standard deviation, respectively. A significant positive correlation was observed between the polymer mole fraction and LC50 (**B**); however, no significant difference was detected among the LC50s calculated for the runs including the six different selected polymers (**C**). R1, R2, …, R23 indicate the “runs” included in the study by Design Expert. Please see Supplementary Table S3 for details.

We estimated the LC50 of the 23 runs in MDA-MB-231 cells using a similar approach and the results are summarized in Figure 10. LipofectamineTM 2000 showed the most toxicity (LC50 = 110.2 nM of siRNA delivered), and the LC50 of R16/DOPE was 186 nM of delivered siRNA, which was slightly lower than the LC50 observed for R16 in the LNP runs (LC50 = 214 nM of siRNA delivered). LipofectamineTM is known to be moderately toxic in many cell lines [34,35]. We have previously reported a higher toxicity (LC50 = 57 nM of siRNA delivered) in MDA-MB-231 cells [36]. Only a few LPNP formulations had a lower LC50 (higher toxicity) than the R16/DOPE LNP, which included runs 9 and 22 (LC50 of 162 and 168 nM of delivered siRNA, respectively). The lowest toxicity was observed for runs 8 and 14 (LC50 of 356 and 328 nM of siRNA delivered). A positive correlation was observed between the polymer mole fraction and LC50 (R = 0.62; *p* value = 0.002), which indicates the relative safety of the selected polymers in the MDA-MB-231 cells (Figure 10B). While P3 and P5 had the lowest and highest LC50 among the LPNPs incorporating different polymers, respectively (221 and 290 nM of siRNA delivered), no significant difference was observed among the six different polymers selected (Figure 10C).

The respond surface plots for the toxicity of the 23 runs are presented in Supplementary Figure S7. The model used for analyzing LC50 data was a quadratic main effects model, which yielded an overall model F-value of 5.97, indicating significance (*p*-value = 0.0034). This result suggests that the selected factors can explain some of the variability in cytotoxicity and that the model successfully captured relevant trends in the response. The ANOVA analysis reveals that the linear mixture effect (F-value = 13.47, *p* = 0.0012) was statistically significant, which means that the interactions between mixture components influenced the cytotoxic response. However, the DE interaction term (F-value = 0.0003, *p* = 0.9856) was not significant, suggesting that additional refinement of the model or inclusion of other factors might improve the ability to capture the complexity of cytotoxicity. This aligns with observations in nanoparticle-based drug delivery studies, where synergistic or antagonistic effects between formulation components often depend on subtle variations in their ratios and structural properties. The model’s fit statistics showed a predicted R^2^ of 0.2383 and an adjusted R^2^ of 0.3557. While the difference between these values is relatively small (less than 0.2), the low predicted R^2^ indicates limited predictive power, implying that the model may not fully generalize to independent data points. Adequate precision was measured at 6.32, above the threshold of 4, which suggests that the model has an adequate signal-to-noise ratio for navigating the design space. A lack of fit test (F-value = 32.37, *p* = 0.0006) was found to be significant, indicating that the model does not fully represent the observed data. This suggests that additional terms or a more complex model may be required to better capture the systematic variability in cytotoxicity. The significant lack of fit may also reflect unaccounted biological factors, such as cellular heterogeneity or differential sensitivity to nanoparticle formulations, which are common challenges in cytotoxicity assays. Please see the summary of statistical analysis in Supplementary Table S4.

### 2.9. Silencing Efficiency of LPNPs in MDA-MB-231 Cells

**Figure 11:**
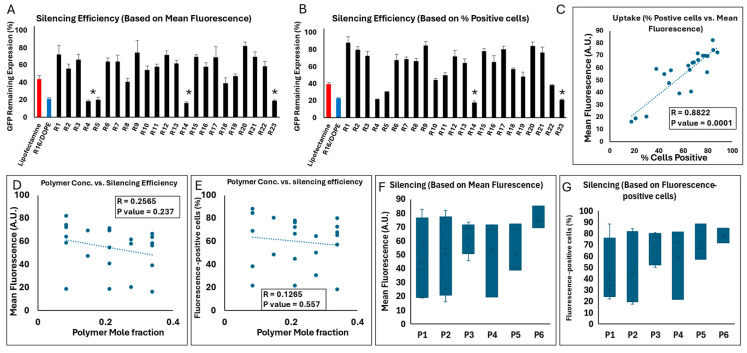
The silencing efficiency of LPNPs for GFP silencing in MDA-231-GFP cells as calculated based on mean fluorescence in the cell population (**A**) and the percentage of cells positive for the GFP fluorescent signal (**B**). Bars and error bars represent the average value (n = 3) and standard deviation, respectively, for the GFP remaining expression (%). The * indicates significant difference with the silencing efficiency observed with R16/DOPE. The mean fluorescence and percentage of fluorescence-positive cells for the two groups with error bars are provided in Supplementary Figure S8. These two indicators had a significant correlation ((**C**); *p* value = 0.0001). A slight negative correlation was observed between the mole fraction of incorporated polymer and the silencing efficiency based on mean fluorescence (**D**) and % based on % GFP positive cells (**E**), which was not statistically significant. Also, the average of silencing efficiency based on mean fluorescence (**F**) and based on percentage of GFP-positive cells (**G**) showed little variability among different polymers. R1, R2, …, R23 indicate the “runs” included in the study by Design Expert. Please see Supplementary Table S3 for details.

Targeting GFP in MDA-231-GFP cells (in comparison to scrambled siRNA) was again used as our approach to investigate the silencing efficiency of LPNPs by quantifying both mean fluorescence and percentage of GFP-positive cells. A significant positive correlation was again observed between the silencing efficiency calculated based on mean fluorescence vs. percentage of GFP-positive cells (R = 0.8822; *p* = 0.0001; Figure 11C). The GFP remaining expression (%) was calculated based on both mean fluorescence and percentage of GFP-positive cells (Figure 11A and Figure 11B, respectively). The individual readings for cells exposed to scrambled or GFP-targeting siRNA are presented in Supplementary Figure S8. Lipofectamine downregulated the GFP signal to ~44% and ~40% based on mean fluorescence and percentage of GFP-positive cells, respectively. R16/DOPE showed a significantly higher efficiency on both accounts (~21 and 23% remaining expression, respectively). All 23 runs showed some level of silencing, with run 20 showing the least efficiency (82 and 84% GFP remaining based on mean fluorescence and % GFP-positive cells, respectively). Runs 1, 9, and 15, 17, and 21 also showed limited efficiency. On the other hand, runs 4, 5, 14, and 23 showed significant silencing efficiency. In fact, run 4 showed a significantly higher silencing efficiency than R16/DOPE in terms of mean fluorescence (*p* value = 0.039), but the difference was not significant when the silencing efficiency was calculated based on % of GFP-positive cells (*p* value = 0.067). Runs 14 and run 23 showed higher efficiency than R16/DOPE on both methods of calculation. Run 14 showed the most downregulation among all the runs (16.3 and 18% based on mean fluorescence and % of GFP-positive cells, respectively), which was statistically significant compared to R16/DOPE (*p* values of 0.015 and 0.022, respectively), but not with run 4 or run 23 (Figure 11A,B). The polymer concentration alone did not correlate significantly with the silencing efficiency calculated based on mean fluorescence (Figure 11D) or based on percentage of GFP-positive cells (Figure 11E). Similarly, one-way ANOVA test did not show a significant difference among the six polymers included in the study in terms of silencing efficiency based on mean fluorescence or % GFP-positive cells (Figure 11F,G, respectively). 

Again, the correlation between the cellular internalization and silencing efficiency achieved by LPNPs was not statistically significant (similar to what we observed for LNPs; Supplementary Figure S9); however, a trend was obvious, and the correlation factors were higher than what was observed for LNPs (R = 0.3697 and 0.3323 for uptake and silencing efficiency based on mean fluorescence and percentage of fluorescent-positive cells, respectively), and the *p* value just missed the significance threshold for the data based on mean fluorescence (*p* value = 0.083). Again, the response surface plots were either based on one factor (the selected polymer) for a pre-determined polymer mole fraction (0.085, 0.2125, or 0.34) or a two-component mix, and the one-factor respond surface plots for silencing efficiency are summarized in the Supplementary Figure S10. The analysis of silencing efficiency based on mean fluorescence for LPNPs revealed strong statistical significance in the quadratic main effects model, with an overall model F-value = 74.78 and *p*-value = < 0.0001, suggesting that the model was able to explain a substantial portion of the variability in silencing efficiency. The model’s adjusted R^2^ value of 0.9820 indicated that about 98.2% of the variability could be explained, reflecting excellent explanatory power. The lack of fit test (F-value = 1.00, *p* = 0.4307) was found to be non-significant, indicating that the model adequately captured the data without evidence of significant lack of fit. Residual analysis further supported the adequacy of the model. 

The coefficients analysis revealed that polymer concentration had significant positive effect on silencing efficiency with the coefficient of 49.85 (95% CI: 47.31 to 52.40) for polymer concentration. This value suggests that increasing the concentrations of polymer significantly enhances silencing efficiency. The interaction between Dlin and polymer concentration (DE) exhibited a slight negative effect (coefficient = −7.02, 95% CI: −17.33 to 3.29), indicating a potential reduction in efficiency when these factors are combined, though the confidence interval suggests uncertainty in this effect. This interaction may reflect competing mechanisms, such as reduced nanoparticle stability or suboptimal endosomal escape efficiency when both components are present in high concentrations. Conversely, specific polymer types demonstrated both positive and negative effects: DF [2] had a strong positive effect (coefficient = 20.37, 95% CI: 14.70 to 26.04), enhancing silencing efficiency, and DF [4] exhibited a strong negative effect (coefficient = −42.17, 95% CI: −47.87 to −36.46), suggesting inhibitory properties in certain formulations.

The analysis of silencing efficiency based on % GFP-positive cells for LPNPs revealed mixed results in terms of model fit and the significance of individual factors. The ANOVA results demonstrated that the quadratic main effects model was statistically significant (F-value = 3.41, *p* = 0.0266), suggesting that the model was able to explain some variability in silencing efficiency. However, the model’s adjusted R^2^ value of 0.6027 indicated that only about 60% of the variability could be explained, reflecting moderate explanatory power. The significant lack of fit (F-value = 45.63, *p* = 0.0004) indicated that the model did not adequately capture the data, highlighting the need for additional or alternative variables to improve accuracy. The *p*-values for individual terms underscored the importance of certain factors, such as the interaction between Dlin and polymer concentration (DF) (*p* = 0.0134), which appeared to have a notable influence on silencing efficiency. However, interaction terms, such as DF [4] (coefficient = −42.09, 95% CI: −69.23 to −14.95), revealed that specific polymer interactions with Dlin could reduce silencing efficiency. Please see the summary of statistical analysis in Supplementary Table S4.

### 2.10. Optimum LPNP Formulation

**Figure 12:**
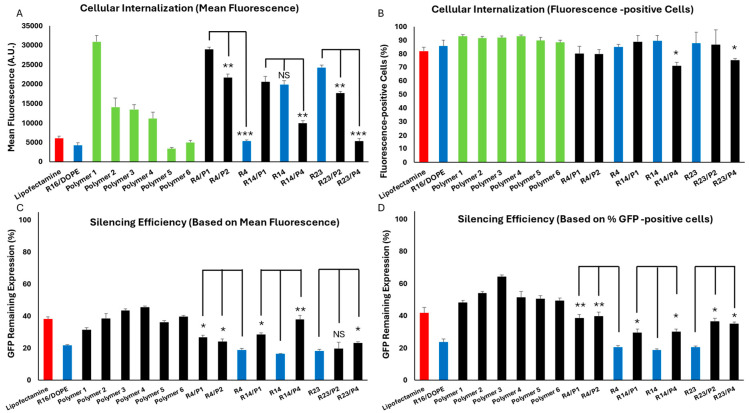
Validation of the best performing LPNPs in comparison to free polymers and variations in their composition. Bars and error bars indicate the average and standard deviation of the results for each group, respectively (n = 3). NS, *, **, and *** represent “not significant” (*p* > 0.05), *p* < 0.05, *p* < 0.005, and *p* < 0.0005, respectively. Cellular internalization in MDA-MB-231 cells was evaluated by mean fluorescence of cell populations (**A**) and percentage of GFP-positive cells (**B**). The silencing efficiency in MDA-231-GFP cells is calculated based on mean fluorescence (**C**) and % GFP-positive cells (**D**).

Design expert analyzed the data presented above and concluded that the best performing formulation was DOPE (0.46)/phosphatidylcholine (0.1)/cholesterol (0.1)/polymer 2 (0.34), which is the exact composition as R14. It is noticeable that the best composition completely replaced Dlin-MC3-DMA with one of the polymers included in the study. We repeated the cell internalization and silencing efficiency experiments in MDA-MB-231 and MDA-231-GFP cells, respectively, to validate the suggested formulation, by including the following study groups: Lipofectamine and R16/DOPE (as controls), polymers 1–6 (to compare the performance of free polymers to LPNPs), R4, R4/P1, and R4/P2 (R4 duplicates with polymers 1 and 2 replacing polymer 4), R14, R14/P1, and R14/P4 (R14 duplicates with polymers 1 and 4 replacing polymer 2), and R23, R23/P2, and R23/P4 (R23 duplicates with polymers 2 and 4 replacing polymer 1). The cellular internalization results are summarized in Figure 12A,B for mean fluorescence and percentage of fluorescence-positive cells, respectively. Among free polymers, polymer 1 created the highest mean fluorescence, which is similar to our observations in LPNP cellular uptake (Figure 9F). Polymers 1–4 created significantly higher mean fluorescence signals compared to Lipofectamine and R16/DOPE. Switching to polymers 1 or 2 in R4 (which originally contained polymer 4) significantly increased the mean fluorescence, which again confirms the higher efficiency of polymers 1 and 2 in cellular internalization compared to polymer 4. This was also obvious in comparing R14 (originally containing polymer 2) to the alternatives containing polymers 1 and 4. Cells exposed to R23 (originally containing polymer 1) showed a higher mean fluorescence than alternatives containing polymers 2 or 4 (Figure 12A). As expected, most of the study groups showed a near maximum percentage of fluorescence-positive cells, except for R14 and R23 alternatives containing polymer 4 (Figure 12B). 

In terms of silencing efficiency calculated based on mean fluorescence, all six free polymers performed similarly to Lipofectamine^TM^, but less efficient than R16/DOPE. When calculated based on percentage of GFP-positive cells, however, polymers 2 and 3 were less efficient than Lipofectamine^TM^ as well, when used free. Interestingly, all three selected original compositions (R4, R14, and R23) showed higher efficiency compared to the alternative created by switching the polymers. All three selected LPNP formulations showed a higher efficiency than R16/DOPE (selected LNP formulation); however, no significant difference was observed among the three LPNP formulations (Figure 12C,D). The mean GFP fluorescence and percentage of GFP-positive cells for the two groups (cells exposed to scrambled siRNA or GFP-targeting siRNA) are provided in Supplementary Figure S11. 

While these side-by-side comparisons did confirm the choices made by algorithm for the selected optimum formulation, this was not the case for the LNP experiments. Finding a more efficient choice than the recommended formulation by algorithm could be at least partially due to the selected CQAs. We decided to analyze the biological outcomes for the designed runs (uptake, silencing efficiency, and toxicity) instead of physiochemical characteristics of the nanoparticles. Based on previous experience, we did not anticipate a significant variation in hydrodynamic diameter (due to the use of mini extruder which by default creates particles with the approximate size of the membrane pores), ζ-potential (except an anticipated increase towards positive values for LPNPs compared to LNPs), or encapsulation efficiency (based on previous experience with a different LNP [14]). We performed a small study to verify this assumption on two LNP formulations (optimal LNP formulation recommended by Design Expert or OF and R16/DOPE) and three LPNP formulations (R4, R14, and R23) which incorporated three different polymers (with different mole fractions) in the nanoparticle composition (Supplementary Table S5). While size and polydispersity were expectedly consistent among all nanoparticles, the ζ-potential of LNPs was negative, and for LPNPs, it was positive. The values of the ζ-potential of the LPNPs perfectly correlated with the mole fraction of the polymer (correlation factor = 0.94), regardless of the chemical structure of the polymers. This data indicate relative consistency in size and encapsulating efficiency of nanoparticles in general, and mole fraction of the polymer as the only factor affecting ζ-potential.

**Figure 13:**
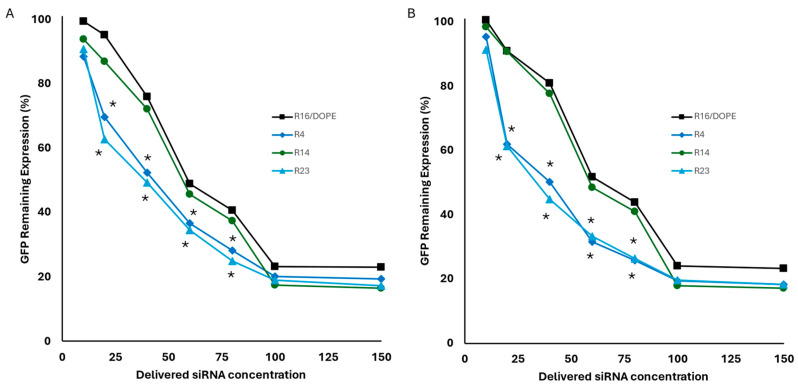
Concentration/response curve for selected LNP (R16/DOPE) and LPNP formulations in MDA-MB-231 cells calculated based on mean fluorescence (**A**) and percentage of GFP-positive cells (**B**). Asterisks indicate significant difference with R16/DOPE silencing efficiency.

While the selected LPNP runs (R4, R14, and R23) performed better than R16/DOPE (selected formulation for LNPs), the difference in silencing efficiency was not overwhelming (unlike internalization efficiency which was clearly higher for the selected LPNP formulations). Therefore, we decided to explore the “dose–response” curve for these formulations to study the silencing efficiency in smaller siRNA concentrations delivered to breast cancer cells. We repeated GFP silencing with a wide array of delivered siRNA concentrations (10, 20, 40, 60, 80, 100, and 150 nM) in addition to the original 100 nM concentration used in our screening studies for selected LNP and LPNP formulations (Figure 13). Among three selected LPNP formulations, R14 showed a similar trend in efficiency to the LNP formulation (R16/DOPE); however, both R4 and R14 showed significantly higher efficiency in lower concentrations (20, 40, 60, and 80 nM) both when calculated based on mean fluorescence (Figure 13A) or percentage of GFP-positive cells (Figure 13B). This is particularly interesting since R14 showed the lowest mean fluorescence in cellular internalization experiments among these three selected formulations (although still significantly higher than R16/DOPE). 

### 2.11. LPNP Performance in Prostate Cancer Cells

**Figure 14:**
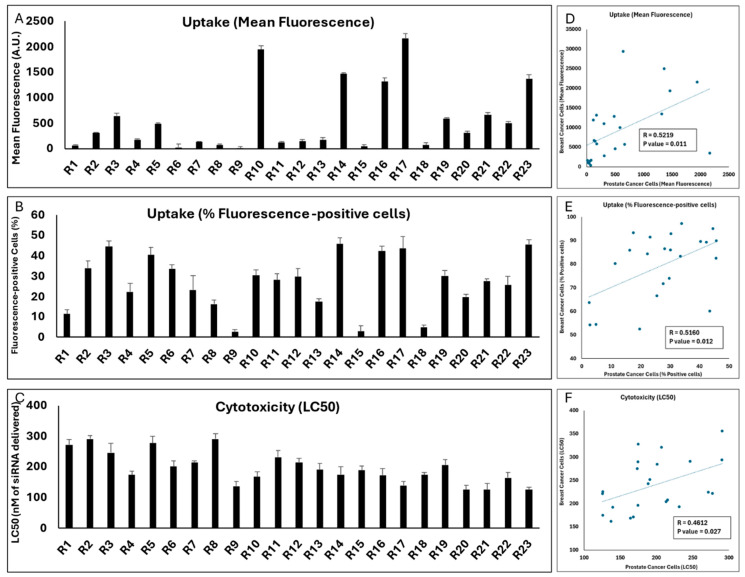
Performance of Design Expert LPNP “runs” in human prostate cancer cell line LNCaP: The cellular internalization, quantified both as the mean fluorescence of the cell population (**A**), and percentage of fluorescence-positive cells (**B**), and cytotoxicity of the runs represented as LC50 calculated based on nM of siRNA delivered (**C**) were investigated in the prostate cancer cells. The correlation graphs, as well as R and *p* values are presented in (**D**), (**E**), and (**F**), respectively.

Similar to LNPs, we also tested the reproducibility of cellular internalization and cytotoxicity of the LPNPs in LNCaP cells, and the results are summarized in Figure 14. The cellular internalization did not reach the levels observed in MDA-MB-231 cells. The mean fluorescence and percentage of fluorescence-positive cells did not exceed 2166 A.U. and 45.7% (both for Run 17), respectively, compared to ~29,400 A.U. and >97% observed in MDA-MB-231 cells. This was similar to what we observed in LNP experiments, which is an indication that LNCaP cells might be a more difficult cell to “transfect” using this approach compared to MDA-MB-231 cells. The LC50 of the 23 runs varied between 125 and 290 nM of siRNA delivered, which was comparable to the range observed in MDA-MB-231 cells (~160 to 356 nM of siRNA delivered). Perhaps most importantly, however, a significant correlation was observed for all three factors investigated between the two cell lines, which further confirmed our observations in LNP experiments. 

## 3. Materials and Methods

### 3.1. Materials

1,2-Dioleoyl-sn-glycero-3-phosphoethanolamine (DOPE; 850725P), Distearoylphosphatidylcholine (DSPC; 850365P), cholesterol (700100P), phosphatidylcholine (95%; 131601P), ALC-0315 (890900O), and Avanti Mini Extruder were acquired from Avanti Polar Lipids, Inc. (Alabaster, AL, USA). Dlin-MC3-DMA (SKU: 25325) was obtained from Nanosoft Biotechnology LLC, (Winston-Salem, NC, USA). Silencer Negative Control siRNA (catalogue no. AM4635) was acquired from Life Technologies (Grand Island, NY, USA). Alexa Fluor 647 (AF647)-siRNA (catalogue no 1027295) and Alexa Fluor 488(AF488)-labeled siRNA (catalogue n. 1027292) were purchased from Qiagen (Germantown, MD, USA). The cell counting 8 (CCK-8) (catalogue no. B34304) kit was provided by Selleck Chemicals LLC (Houston, TX, USA). Cell culture reagents, plates, phosphate buffer saline (PBS), and consumables were purchased from VWR (Radnor, PA, USA). The structure of the low-molecular-weight, hydrophobically-modified PEIs are shown in Figure 8 and were reported before: (i) the polymer P1 in [37], (ii) the polymer P2 and P3 in [38], (iii) the polymer P4 in [39], and (iv) the polymers P5 and P6 in [40]. Green Fluorescence Protein (GFP)-siRNA (sense sequence: 5′-GAACUUCAGGGUCAGCUUGCCG-3′) was obtained from Integrated DNA Technologies Inc. (Coralville, IA, USA).

### 3.2. Nanoparticle Composition

For this study, we used the Design Expert software that provides an avenue for systematic evaluation of the different variables in a multicomponent formulation. This approach based on response surface methodologies facilitates an optimization process with a limited number of experimental formulations. In this approach, we needed to define (i) the Critical Process Parameters (CPPs), which are the input variables in the compositions; (ii) the Critical Quality Attributes (CQAs), which are the results of the experiments performed on characterizing and assessing the performance of each formulation (also referred to as “run”); and (iii) the model, which is the mathematical equation that correlates the selected CPPs to each of the CQAs [41]. 

The key components of our nanoparticle formulations included are as follows:

- Phospholipids
  - 1,2-distearoyl-sn-glycero-3-phosphocholine (DSPC): One of the building blocks of cell membrane, which is sometimes referred to as “helper lipid” [42]. It has been a consistent component of FDA-approved LNPs patisiran (Onpattro^®^, Alnylam Pharmaceuticals, Inc., Cambridge, MA, USA), tozinameran (Comirnaty^®^; COVID-19 vaccine, Pfizer (New York, NY, USA)/BioNTech (Mainz, Germany)), and elasomeran (Spikevax; COVID-19 vaccine, Moderna, Cambridge, MA, USA) [32];
  - 1,2-dioleoyl-sn-glycero-3-phosphoethanolamine (DOPE): A synthetic phospholipid that has been reported to promote fusion with endosomes [43]. We selected DOPE as an alternative to DSPC despite the consistent appearance of DSPC in FDA-approved LNP, mostly due to the promising results in siRNA delivery we have experienced with this phospholipid in our lab [14,44,45];
  - Phosphatidylcholine (PC): Employed to enhance the fluidity and stability of the nanoparticle membrane.
- Ionizable Lipids
  - ALC-0315: A synthetic ionizable amino lipid with a distinct cone-shaped molecular structure with lipid tale branching [46] that is used in the composition of tozinameran (Pfizer/BioNTech). ALC-0315 recently showed promising result in siRNA delivery in vivo [26] and in a Design-of-Experiment comparison for self-amplifying (saRNA) delivery [25];
  - Dlin-MC3-DMA: Another synthetic ionizable amino lipid with pKa of approximately 9.4 [46] and one ester linker (as opposed to two in ALC-0315) that has been used in patisiran formulation. It was included in the study as another commonly used ionizable lipid in LNP composition.
- Sterol
  - Cholesterol: Incorporated to stabilize the lipid layers and improve the structural integrity of the nanoparticles.
- Polymers
  - Six different polymer structures were included in this study. These polymers have been investigated extensively in Dr. Uludag’s lab for their efficiency in forming complexes with siRNA and delivery efficiency in different cancer types [39,40,47]. The polymers were selected to cover a wide variety of hydrophobic moieties and electrical charge, and the structures are illustrated in Figure 8. Our hypothesis is that the optimum polymer would represent the optimal balance between these two variables that can maximize the serum stability and cellular internalization.

### 3.3. Experimental Design Using Design Expert

To explore the LNP compositions, we employed Design Expert software with the CPPs summarized in Table 2. This approach facilitates the investigation of multicomponent formulations by analyzing the data collected from a limited number of formulations (“runs”) and drawing conclusions on optimum formulation components. The range of the mole fractions for each of these components in the Design Expert input was selected based on literature and our previous experiments. 

This software facilitated the use of optimal combined designs and response surface methodologies, enabling us to systematically vary the composition of the nanoparticles and assess the impact on the defined CQAs: cytotoxicity, siRNA cellular internalization, and gene silencing efficacy. Supplementary Table S1 summarizes the 29 experimental “runs” designed using the Design Expert software, representing the variations in the formulation components. 

In preparation of LPNPs, we used the best performing composition for the LNPs as the starting point, and only had two CPPs for incorporating the polymers into the nanoparticles: the mole fraction of polymer and the polymer structure. The range for mole fraction of the polymer was 0.085–0.34 (zero was not included in the range, since a composition without the polymer would have been the selected LNP). The mole fraction of 0.34 represented formulations in which the ionizable lipid was completely replaced by the polymer.

In this study, we utilized an “optimal combined design” as the selected model to address the constraints of maintaining a total mole fraction of 1 for the variable excipients. This approach efficiently managed the interplay between numeric CPPs (e.g., concentrations of excipients) and categorical CPPs (e.g., types of excipients). The design ensured compliance with the mole fraction constraint by systematically varying excipient levels within the defined boundaries. This method maximized experimental efficiency and improved the robustness and reproducibility of the formulation, ultimately enhancing the accuracy of our statistical models and leading to an optimized drug formulation.

### 3.4. Nanoparticle Formation 

LNPs and LPNPs were prepared using a mini extruder (Avanti Polar Lipids, Alabaster, AL) and 100 nm membranes. The siRNA alone (for LNPs) or siRNA and polymer (for LPNPs) were mixed in the aqueous phase. All the lipid components (phospholipids, ionizable lipid, sterol) were added to ethanol, and the ratio of ethanol/aqueous phases was 1:3 in all formulations. The two phases were loaded in two different glass syringes and were mixed through the mini extruder, passing the 100 nm membrane 50 times. The nanoparticles were then transferred to a dialysis bag with a molecular weight cut-off of 500–1000 Daltons (Biotech CE dialysis tubing), which was placed in a beaker full of purified water for 15 min to remove the ethanol from the colloidal dispersion. In our previous studies, the nanoparticles prepared with this method had an average size of ~100 nm and were free of ethanol [14]. We frequently used Zetasizer ZEN3600 (Malvern, Worcestershire, UK) to monitor the size of the particles pre- and post-dialysis and did not observe significant changes in size or polydispersity.

### 3.5. Cell Line Models 

Breast cancer: MDA-MB-231 triple negative human breast adenocarcinoma cells (ATCC HTB-26) and MDA-MB-231 cells permanently expressing Green Fluorescence Protein (MDA-231-GFP; kindly provided by Dr. Judith Hugh at the Faculty of Medicine and Dentistry, University of Alberta) were thawed and cultured in Dulbecco’s Modified Eagle’s Medium (DMEM)—low glucose. Medium was supplemented with 100 U/mL penicillin, 100 µg/mL streptomycin and 10% v/v fetal bovine serum (FBS) and cells were incubated in 37 °C and 5% CO_2_ at all times. For expansion, cells were trypsinized using 0.05% trypsin when 80–90% confluent and were resuspended in fresh medium after centrifuge at 600 RPM for 5 min. 

The fluorescent signal in MDA-231-GFP cells was monitored consistently on every experiment day to validate stable GFP expression. The mean fluorescence of the cell population and the percentage of GFP-positive cells were both stable at 14,034 ± 932 AU and 85.2 ± 2.1%, respectively.

Prostate cancer: Androgen-sensitive human prostate cancer cell line LNCaP was cultured at 80% confluency using RPMI 1640 medium (Mediatech, Inc., New York, NY, USA) containing 1X L-glutamine, 10% FBS, and 1% penicillin/streptomycin. Cells were trypsinized with 0.05% trypsin for 2 min and were resuspended in fresh medium after being at 600 rpm for 5 min. Thirty percent of the cell population was transferred to a new culture dish with fresh media. The incubation conditions were similar to the breast cancer cells. 

The prostate cancer cell line was added to evaluate the performance of nanoparticles in a different cancer type.

### 3.6. Cytotoxicity Assay

MDA-MB-231 or LnCaP cells were cultured in a 96-well plate until they reached 50% confluency. Subsequently, the cells were exposed in triplicate to the following: No treatment (NT; normal saline), and depending on the study, LNPs or LPNPs delivering final concentrations of 25, 50, 75, 100, or 200 nM siRNA. The cells were then incubated at 37 °C in 5% CO_2_ for 48 h. Cell viability was assessed using a Cell Counting Kit-8 (CCK-8) which utilizes WST-8. Briefly, 10 μL of CCK-8 reagent was added to each well. After an additional 2 h of incubation at 37 °C, the optical density was measured at 450 nm with a SpectraMAX M5 UV–Vis Plate Reader (Molecular Devices, San Jose, CA, USA). Three wells containing only growth medium were included in every plate and the average reading was used as blank (subtracted from all other readings). The average of the reading for the untreated cells was assumed as 100%, upon which all the results for “treated cells” were normalized and expressed as percent of viable cells as an indication for the cytotoxic effect of the LNPs and LPNPs. Lethal concentration for 50% cell death (LC50) was calculated based on the graphs where siRNA delivered concentration was plotted against the percent of viable cells.

### 3.7. Cellular Internalization of Nanoparticles

Flow Cytometry. MDA-MB-231 or LnCaP cells were exposed to one of the following study groups: Normal saline (no treatment; NT), free AF647-siRNA, AF647-labeled siRNA delivered by LNPs or LPNP formulations, and AF647-labeled siRNA delivered by Lipofectamine^TM^ 2000 (Thermo Fisher Scientific, Waltham, MA, USA) as a commonly used commercially available reagent for in vitro siRNA delivery. The final siRNA concentration in the well was 36 nM. The cells were seeded in 24-well plates, and once 70% confluency was reached, study groups were added to the cell culture in triplicate wells. Lipofectamine^TM^ was used according to the manufacturer’s guidelines. Briefly, Lipofectamine^TM^ and siRNA were diluted in antibiotic-free OPTIMEM medium in separate tubes and were mixed. After 10 min of incubation at ambient temperature, the cell culture medium was removed from wells, and the siRNA complexes were added to the wells in OPTIMEM. After 4 h of incubation at 37 °C, the complexes were removed, and the cell culture medium was added to the wells. 

After 24 h incubation at 37 °C and 5% CO_2_, the media was removed, and the cells were detached using 0.05% trypsin. The cells were fixed using 3.7% formaldehyde in PBS and transferred to a 96 well plate. Each sample was evaluated using a FACSVERSE flow cytometer (BD Biosciences; San Jose, CA, USA). Following each flow cytometry analysis, the percentage of cells positive for fluorescence signal and the mean fluorescence of the cell population was calculated using the calibration of the signal gated with NT cells in order to obtain an autofluorescence of approximately 1% of the population in “no treatment” group.

Confocal Microscopy. In order to visually confirm the cellular internalization of nanoparticles, MDA-MB-231 cells were exposed to one of the following study groups: free AF488-siRNA, AF488-labeled siRNA delivered by Lipofectamine^TM^ 2000, optimum formulation, and selected formulations. We used Alexa Fluor 488 (which is a green fluorescent dye) for this experiment due to the red dye used to stain the cell membrane. Sterilized coverslips were placed in every well in 6-well plates and were submerged in 10% FBS in DMEM. The plates were incubated for 30 min at 37 °C to boost the cell adherence to the surface of the coverslips. The FBS/medium was removed then, and MDA-MB-231 cells were seeded on the coverslips. When the wells were 70% confluent, the study groups were added to the wells. Plates were incubated at 37 °C and 5% CO_2_ for 24 h, after which the media was removed. Cells were washed with 1× PBS three times and were fixed using 3.7% formaldehyde in PBS for 10 min. The cells were then rinsed three times with PBS for 5 min. Texas Red Phalloidin solution (40 μL and 10 mg BSA in 10 mL PBS) was added to the cells to stain the cell membrane, and cells were incubated at room temperature for one hour. The cells were then washed with PBS 3 times for 5 min each. To stain the nucleus, one drop of DAPI was added to each slide and the coverslips were stored overnight away from light to dry. The imaging was performed by a Nikon A1R high-definition resonant scanning confocal microscope (Nikon, New York, NY, USA) and NIS-Elements software (AR 4.30.02, 64bit).

### 3.8. Green Fluorescence Protein Silencing 

This experiment was only performed in the GFP-expressing MDA-MB-231 cells. GFP was selected as the model protein for silencing efficiency studies due to the possibility of streamlining a quantitative analysis for silencing efficiency, which is required for the performance of the selected software and the analytical approach. GFP-expressing MDA-MB-231 cells were seeded in 6-well plates in triplicates. After reaching 50% confluency, cells were treated with one of the following groups: no treatment (NT; exposed to normal saline), scrambled siRNA or siRNA targeting GFP delivered by nanoparticles and Lipofectamine^TM^ 2000. The final siRNA concentration in the wells was 100 nM. The cells were incubated at 37 °C and 5% CO_2_ for 48 h. The same FACSVERSE flow cytometer was used to examine and quantify the fluorescent signal and percentage of the cells positive with fluorescent signal in different study groups. The silencing efficiency was calculated based on the remaining expression using the following equation:

$$Remaining\;Expression\;\left(\%\right)=\frac{GFP\;signal\;after\;exposure\;to\;GFP\;targeting\;siRNA}{Average\;of\;GFP\;signal\;after\;exposure\;to\;scrambled\;siRNA}\times 100$$

The use of this equation normalizes the change in *GFP signal* based on any potential changes in the *GFP signal* due to off-target effect, which is a possibility, especially with high *siRNA* concentrations delivered.

### 3.9. Statistical Analysis

The manual direct comparisons were carried out by Students’ *t*-test (non-paired, two-tailed, assuming unequal variance) and correlation factor (R). However, the main statistical analysis was performed by the Design Expert software, using a reduced “quadratic main effects” model to evaluate the main effects and interaction effects of CQAs. The software decided on the significance of the model for each CQA using ANOVA (*p* value < 0.05 was considered significant), and analyzed linear mixture terms (to evaluate the effect of CPPs on the selected CQAs), interaction effects (*p* value < 0.05 was considered significant), lack of fit (*p* value < 0.05 was considered a fit), residual errors, adjusted R^2^ (indicating the fraction of variability explained by the model), and coefficient estimates. Residual analysis by the software provided insight into the appropriateness of the model.

## 4. Conclusions

In this study, we used Design Expert Algorithm to create lipid–polymer nanoparticles (LPNP) and compare them to LNPs optimized by the same algorithm. While the composition recommended by the algorithm as the optimal formulation for siRNA delivery and GFP silencing in MDA-MB-231 cells was confirmed for the lipid selections (DOPE over DSPC, and Dlin-MC3-DMA over ALC-0315), a side-by-side comparison revealed a more efficient alternative formulation.

Incorporating specifically designed polymers into the nanoparticles did enhance the efficiency of internalizing siRNA in this cell line, and three compositions also showed a higher silencing efficiency compared to the selected LNP, especially in lower siRNA concentrations delivered. This might be at least partially due to the enhanced cellular internalization observed with LPNPs, which in turn might be due to enhanced interaction with cell membrane and more efficient endosomal escape. The effect of polymers on the endosomal escape needs to be confirmed in the future studies by using endosomal colocalization imaging. Additionally, the flexibility of polymer structures can be proven especially beneficial in expanding the applications of these nanoparticles and enhancing targeting characteristics. While this study has limitations (such as lack of experiments exploring the mechanisms of internalization, rate of siRNA release, or endosomal escape), we conclude that polymers can be a viable option in enhancing the performance of LNPs in nucleic acid delivery. We also suggest that while formulation algorithms could be very useful in designing multicomponent nanoparticles, the outcomes should be examined closely, and the effect of all the CPPs should be considered. In addition to expanding the in vitro studies to investigate silencing relevant proteins in cancer biology and the effect on cell proliferation, future plans include investigation of the stability of nanoparticles, further in vitro characterizations, delivering other nucleic acids (e.g., CRISPR/as9 and mRNA), addition of targeting moieties for active targeting, exploring new and interesting applications other than cancer therapy (e.g., delivering siRNA as an antivirus strategy), and in vivo animal studies.

## Figures and Tables

**Table 1:** The study design for direct validation of the proposed optimum formulation (OF) against the most efficient “runs” in silencing GFP including the variations of the ionizable lipid and phospholipid I (PC: phosphatidylcholine; Chol.: cholesterol).

LNP Formulation	DOPE	DSPC	PC	Chol.	ALC-0315	Dlin-M3-DMA
Proposed OF	0.4	0	0.1	0.1	0	0.4
OF/DSPC	0	0.4	0.1	0.1	0	0.4
OF/ALC-0315	0.4	0	0.1	0.1	0.4	0
Run 16	0	0.46	0.1	0.1	0	0.34
Run16/DOPE	0.46	0	0.1	0.1	0	0.34
Run 16/ALC-0315	0	0.46	0.1	0.1	0.34	0
Run 26	0	0.55	0.25	0.1	0.1	0
Run 26/DOPE	0.55	0	0.25	0.1	0.1	0
Run 26/Dlin-MC3-DMA	0	0.55	0.25	0.1	0	0.1

ALC-0315: 6-((2-hexyldecanoyl)oxy)-N-(6-((2-hexyldecanoyl)oxy)hexyl)-N-(4-hydroxybutyl)hexan-1-aminium; Dlin-MC3-DMA: Dilinoleyl-methyl-4-dimethylaminobutyrate MC3; DOPE: 1,2-dioleoyl-sn-glycero-3-phosphoethanolamine; DSPC: 1,2-distearoyl-sn-glycero-3-phosphocholine; LNP: lipid nanoparticle; OF: optimum formulation (proposed by the algorithm).

**Table 2:** LNP formulation components and ranges.

LNP Components	Option (s)	Range of Mole Fraction
Phospholipid I	DSPC	0.35–0.6
	DOPE	0.35–0.6
Phospholipid II	Phosphatidylcholine	0.1–0.3
Ionizable lipid	ALC-0315	0.1–0.4
	Dlin-MC3-DMA	0.1–0.4
Sterol	Cholesterol	0.1–0.25

ALC-0315: 6-((2-hexyldecanoyl)oxy)-N-(6-((2-hexyldecanoyl)oxy)hexyl)-N-(4-hydroxybutyl)hexan-1-aminium; Dlin-MC3-DMA: Dilinoleyl-methyl-4-dimethylaminobutyrate MC3; DOPE: 1,2-dioleoyl-sn-glycero-3-phosphoethanolamine; DSPC: 1,2-distearoyl-sn-glycero-3-phosphocholine.

## Data Availability

The original contributions presented in this study are included in the article/Supplementary Material. Further inquiries can be directed to the corresponding authors.

## Supplementary Materials

The following supporting information can be downloaded at https://www.mdpi.com/article/10.3390/ph18060864/s1. Figure S1: Investigating the effect of individual CPPs on the internalization of siRNA in MD-MB-231 cells; Figure S2: The effect of individual CPPs on the toxicity of the LNPs (based on LC50 calculated for the siRNA delivered in nM) in MD-MB-231 cells; Figure S3: The mean fluorescence and percentage of cells positive for GFP fluorescence for MDA-MB-GFP cells exposed to LNPs; Figure S4: Correlation between the uptake and silencing efficiency for LNPs; Figure S5: The effect of individual CPPs on the silencing efficiency against green fluorescent protein (GFP) in MD-MB-231 cells; Figure S6: The one factor respond surface plots for cellular internalization; Figure S7: The one factor respond surface plots for cytotoxicity; Figure S8: The mean fluorescence and percentage of cells positive for GFP fluorescence for MDA-231-GFP cells exposed to LPNPs; Figure S9: Correlation between the uptake and silencing efficiency for LPNPs; Figure S10: The one factor respond surface plots for silencing efficiency; Figure S11: The mean fluorescence (A) and percentage of cells positive for GFP fluorescence for side-by-side comparison of LPNPs; Table S1: The 29 experimental “runs” designed using the Design-Expert software to optimize the composition of Lipid Nanoparticles (LNPs); Table S2: The statistical analysis data for the 29 LNP runs; Table S3: The 28 experimental “runs” designed using the Design-Expert software to optimize the composition of Lipid/Polymer Nanoparticles (LPNPs); Table S4: The statistical analysis data for the 23 LPNP runs; Table S5: Hydrodynamic diameter, polydispersity (PD), and ζ-potential of selected LNP.
