# Supplementary material for: Engineering Lipid–Polymer Nanoparticles for siRNA Delivery to Cancer Cells
Source: Pharmaceuticals (Basel). 2025 Jun 10;18(6):864. doi: 10.3390/ph18060864 (PMC12196244; doi:10.3390/ph18060864)
Supplement: Supplementary file 1 [file pharmaceuticals-18-00864-s001.zip › pharmaceuticals-3637582-supplementary.pdf]

Engineering Lipid-Polymer Nanoparticles for siRNA Delivery to Cancer cells

Supplementary Information

Supplementary Table S1. The 29 experimental "runs" designed using the Design-Expert software to optimize the composition of Lipid Nanoparticles (LNPs)

Runs	DOPE	DSPC	Phosphatidylcholine	Cholesterol	ALC-0315	Dlin-MC3-DMA
1	0	0.35	0.3	0.25	0.1	0
2	0.36	0	0.17	0.25	0.22	0
3	0	0.5	0.1	0.22	0.18	0
4	0.6	0	0.1	0.1	0.2	0
5	0	0.6	0.12	0.1	0.18	0
6	0	0.42	0.3	0.1	0.18	0
7	0	0.37	0.19	0.14	0.3	0
8	0	0.43	0.21	0.24	0	0.12
9	0.36	0	0.1	0.14	0.4	0
10	0.46	0	0.13	0.16	0	0.25
11	0	0.35	0.22	0.1	0	0.33
12	0.52	0	0.2	0.18	0.1	0
13	0.55	0	0.25	0.1	0	0.1
14	0	0.38	0.3	0.13	0	0.19
15	0.35	0	0.18	0.1	0	0.37
16	0	0.46	0.1	0.1	0	0.34
17	0	0.5	0.1	0.1	0.3	0
18	0.52	0	0.2	0.18	0.1	0
19	0.35	0	0.3	0.23	0	0.12
20	0.46	0	0.13	0.16	0	0.25
21	0	0.35	0.1	0.23	0	0.32
22	0.46	0	0.13	0.16	0	0.25
23	0	0.43	0.21	0.24	0	0.12
24	0	0.37	0.19	0.14	0.3	0
25	0.39	0	0.3	0.1	0.21	0
26	0	0.55	0.25	0.1	0.1	0
27	0	0.6	0.13	0.16	0	0.11
28	0.46	0	0.16	0.1	0.28	0
29	0.55	0	0.1	0.25	0	0.1

Supplementary Table S2. The statistical analysis data for the 29 LNP runs

Response	F-value	p-value	Sum of Squares (SS)	Mean Square (MS)	Adjusted R^2	Predicted R^2	Adequate Precision	Lack of Fit
Uptake (% fluorescence-positive cells)	11.57	<0.0001	17.72	0.98	0.7905	0.6957	11.978	0.4809
Uptake (Mean Fluorescence)	5.06	0.0017	29.05	1.38	0.504	0.2485	6.918	0.1275
LC50 (based on nM of siRNA delivered)	3.37	0.0137	0.0001	6.78E-06	0.4918	0.0942	8.13	0.6534
Silencing (based on % GFP-positive cells)	2.21	0.0696	59.5	3.13	0.2803	-0.5094	6.1625	0.0544
Silencing (based on Mean fluorescence)	2.53	0.0469	120.69	5.75	0.2768	-0.0281	5.6427	0.2053

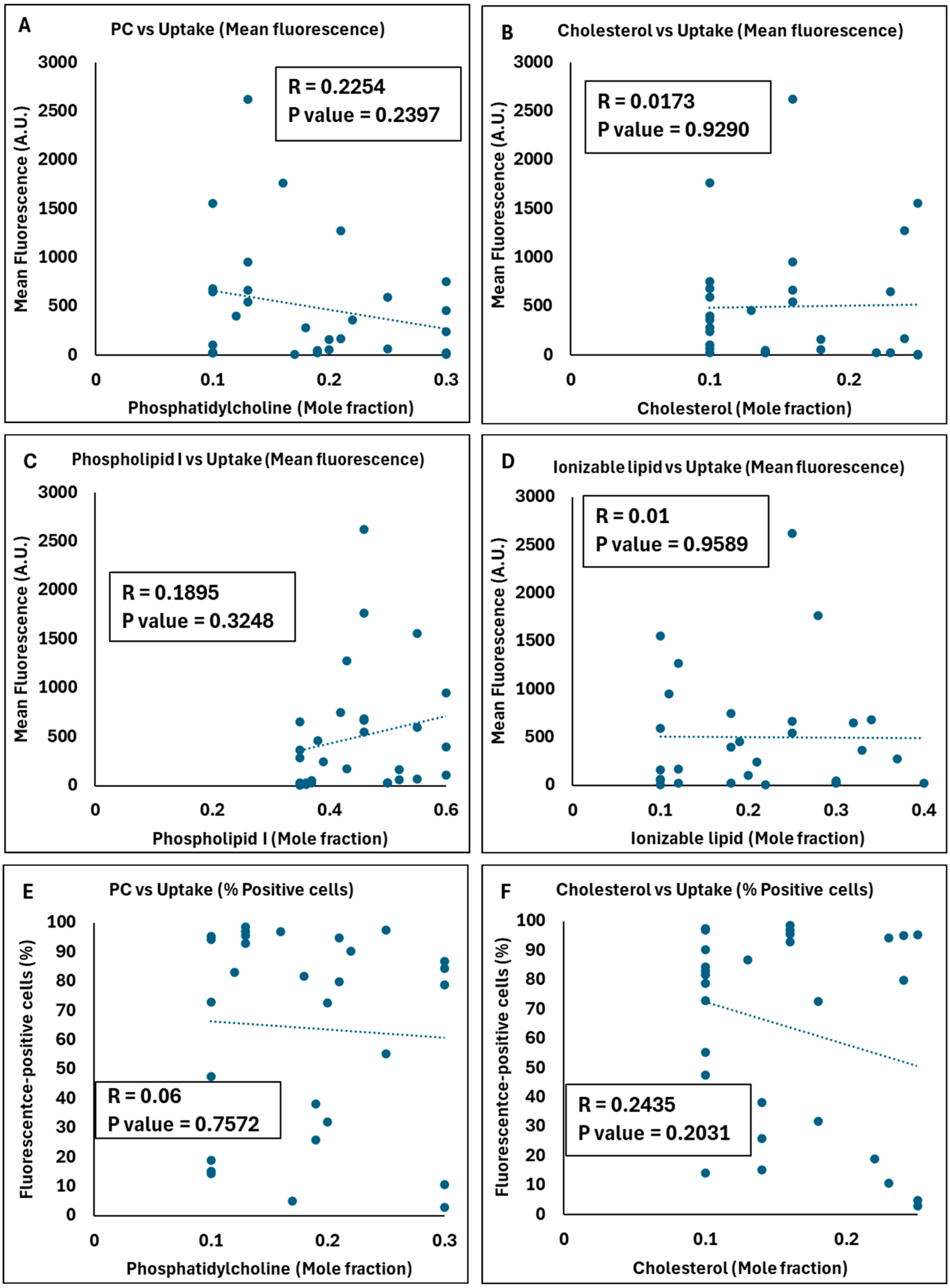
A
PC vs Uptake (Mean fluorescence)
R = 0.2254
P value = 0.2397
Mean Fluorescence (A.U.)
Phosphatidylcholine (Mole fraction)
B
Cholesterol vs Uptake (Mean fluorescence)
R = 0.0173
P value = 0.9290
Mean Fluorescence (A.U.)
Cholesterol (Mole fraction)
C
Phospholipid I vs Uptake (Mean fluorescence)
R = 0.1895
P value = 0.3248
Mean Fluorescence (A.U.)
Phospholipid I (Mole fraction)
D
Ionizable lipid vs Uptake (Mean fluorescence)
R = 0.01
P value = 0.9589
Mean Fluorescence (A.U.)
Ionizable lipid (Mole fraction)
E
PC vs Uptake (% Positive cells)
R = 0.06
P value = 0.7572
Fluorescentce-positive cells (%)
Phosphatidylcholine (Mole fraction)
F
Cholesterol vs Uptake (% Positive cells)
R = 0.2435
P value = 0.2031
Fluorescentce-positive cells (%)
Cholesterol (Mole fraction)

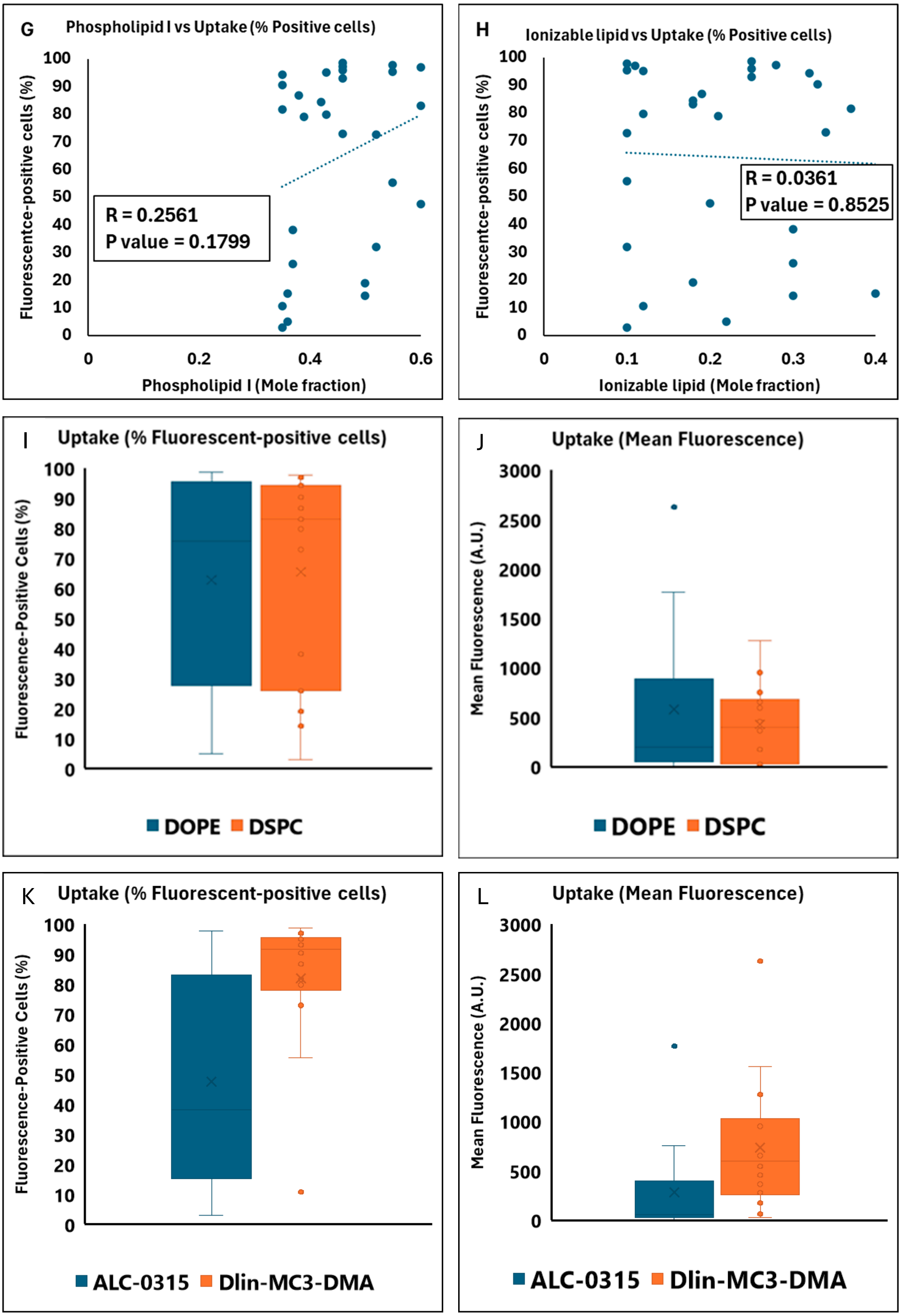
G
Phospholipid I vs Uptake (% Positive cells)
Fluorescentce-positive cells (%)
100
90
80
70
60
50
40
30
20
10
0
0
0.2
0.4
0.6
Phospholipid I (Mole fraction)
R = 0.2561
P value = 0.1799
H
Ionizable lipid vs Uptake (% Positive cells)
Fluorescentce-positive cells (%)
100
90
80
70
60
50
40
30
20
10
0
0
0.1
0.2
0.3
0.4
Ionizable lipid (Mole fraction)
R = 0.0361
P value = 0.8525
I
Uptake (% Fluorescent-positive cells)
Fluorescence-Positive Cells (%)
100
90
80
70
60
50
40
30
20
10
0
DOPE
DSPC
J
Uptake (Mean Fluorescence)
Mean Fluorescence (A.U.)
3000
2500
2000
1500
1000
500
0
DOPE
DSPC
K
Uptake (% Fluorescent-positive cells)
Fluorescence-Positive Cells (%)
100
90
80
70
60
50
40
30
20
10
0
ALC-0315
Dlin-MC3-DMA
L
Uptake (Mean Fluorescence)
Mean Fluorescence (A.U.)
3000
2500
2000
1500
1000
500
0
ALC-0315
Dlin-MC3-DMA

Supplementary Figure S1. Investigating the effect of individual CPPs on the internalization of siRNA in MD-MB-231 cells: Correlation between the mean fluorescence in the cell population with mole fraction of phosphatidylcholine (PC; **A**), cholesterol (**B**), Phospholipid I (**C**), and Ionizable lipid (**D**); Correlation between percentage of fluorescence-positive cells with mole fraction of phosphatidylcholine (PC; **E**), cholesterol (**F**), Phospholipid I (**G**), and Ionizable lipid (**H**); direct comparison of percentage of fluorescence-positive cells for DOPE vs DSPC (**I**) and ALC-0315 vs Dlin-MC3-DMA (**K**), and mean fluorescence for DOPE vs DSPC (**J**) and ALC-0315 vs Dlin-MC3-DMA (**L**). No significant correlation was observed for any of the mole fractions and the indicators of the cellular internalization. There was no significant difference between the percentage of fluorescence-positive cells (P value = 0.838) or mean fluorescence (P value = 0.536) for runs containing DOPE vs the runs containing DSPC. However, the runs containing Dlin-MC3-DMA showed a significantly higher percentage of fluorescence-positive cells compared to runs with ALC-0315 (P value = 0.004). A similar trend was observed for mean fluorescence; however, the difference was not significant (P value = 0.053).

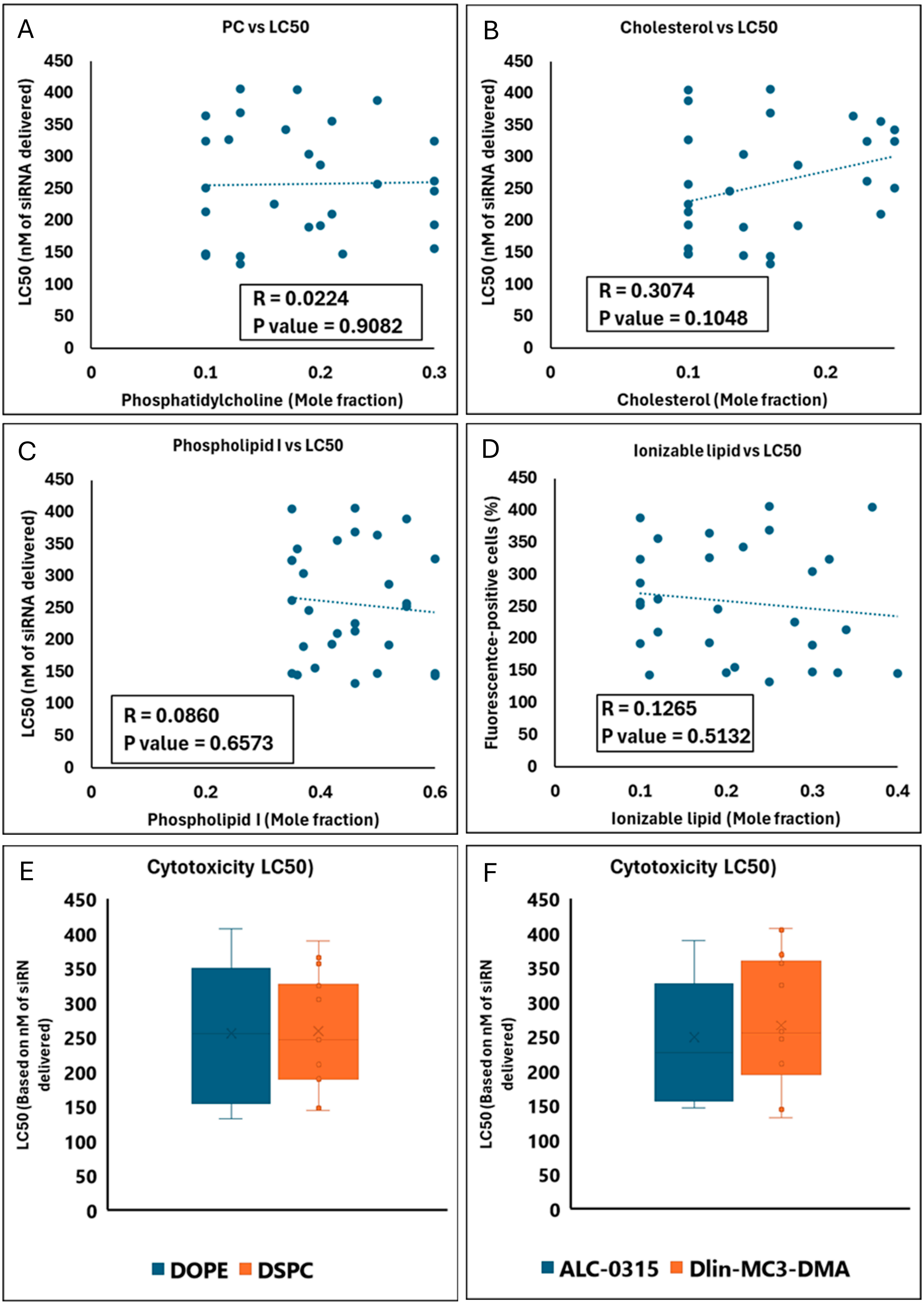

A
PC vs LC50
LC50 (nM of siRNA delivered)
Phosphatidylcholine (Mole fraction)
R = 0.0224
P value = 0.9082
B
Cholesterol vs LC50
LC50 (nM of siRNA delivered)
Cholesterol (Mole fraction)
R = 0.3074
P value = 0.1048
C
Phospholipid I vs LC50
LC50 (nM of siRNA delivered)
Phospholipid I (Mole fraction)
R = 0.0860
P value = 0.6573
D
Ionizable lipid vs LC50
Fluorescentce-positive cells (%)
Ionizable lipid (Mole fraction)
R = 0.1265
P value = 0.5132
E
Cytotoxicity LC50)
LC50 (Based on nM of siRN delivered)
DOPE
DSPC
F
Cytotoxicity LC50)
LC50 (Based on nM of siRN delivered)
ALC-0315
Dlin-MC3-DMA

Supplementary Figure S2. Investigating the effect of individual CPPs on the toxicity of the LNPs (based on LC50 calculated for the siRNA delivered in nM) in MD-MB-231 cells: Correlation between the LC50 with mole fraction of phosphatidylcholine (PC; A), cholesterol (B), Phospholipid I (C), and Ionizable lipid (D); and direct comparison of the LC50s for LNPs containing DOPE vs DSPC (I) and ALC-0315 vs Dlin-MC3-DMA (K). No significant correlation was observed for any of the mole fractions and the toxicity in this cell line. There was no significant difference between the LC50s for runs containing DOPE vs the runs containing DSPC (P value = 0.932) or runs containing Dlin-MC3-DMA vs runs with ALC-0315 (P value = 0.621).

A

B

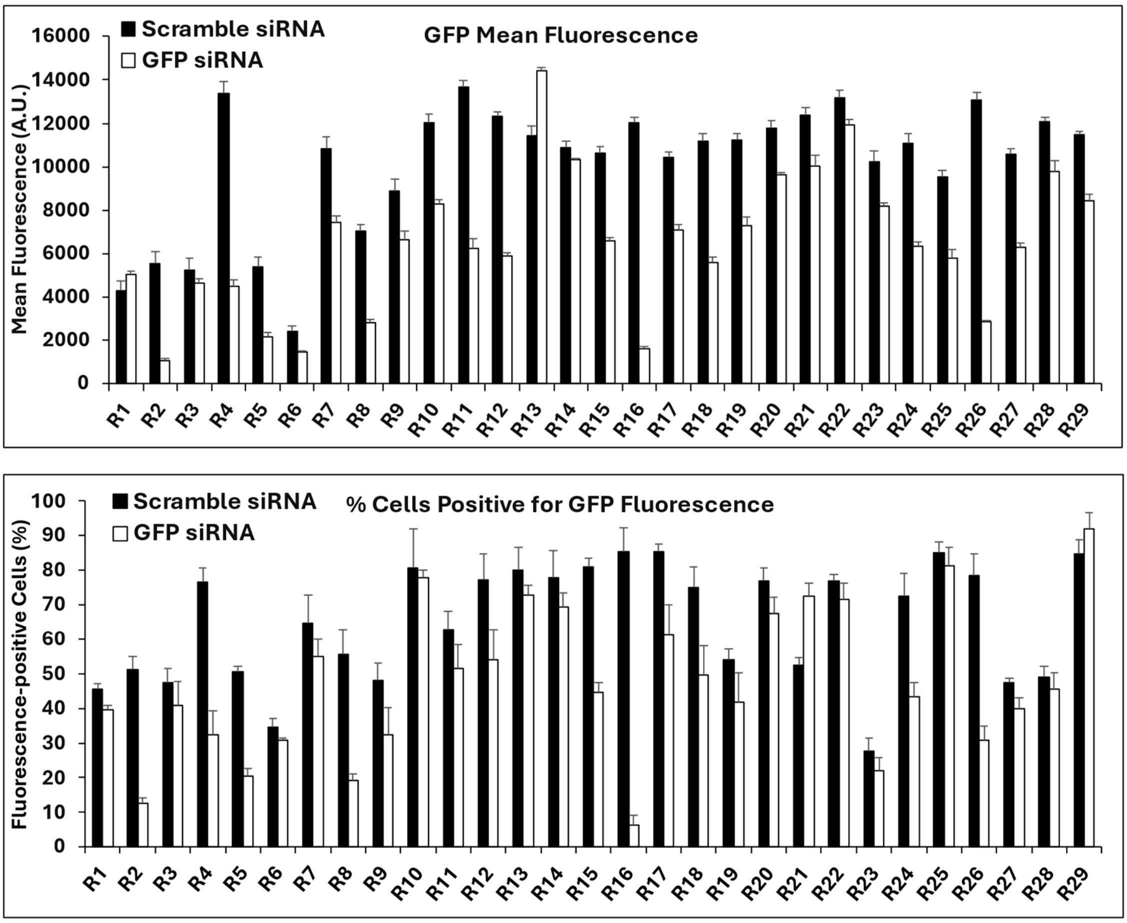

Supplementary Figure S3. The mean fluorescence (**A**) and percentage of cells positive for GFP fluorescence (**B**) for MDA-MB-GFP cells exposed to scrambled or GFP targeting siRNA (100 nM final concentration) delivered by the 29 runs included in the study.

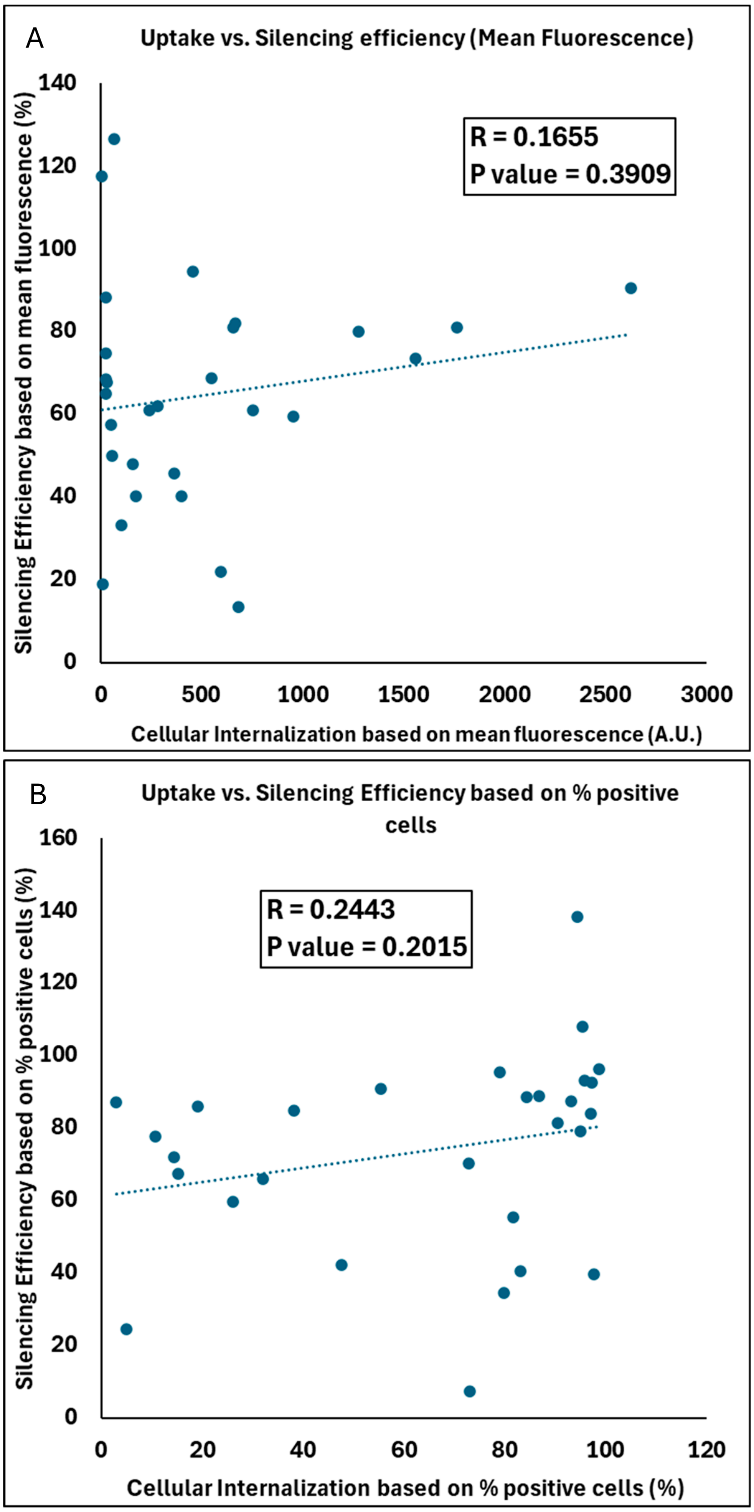

Supplementary Figure S4. Correlation between the uptake and silencing efficiency of the designed 29 runs in MDA-MB-231 and MDA-MB-GFP cells, respectively, as indicated by mean fluorescence (**A**) and percentage of fluorescence positive cells (**B**).

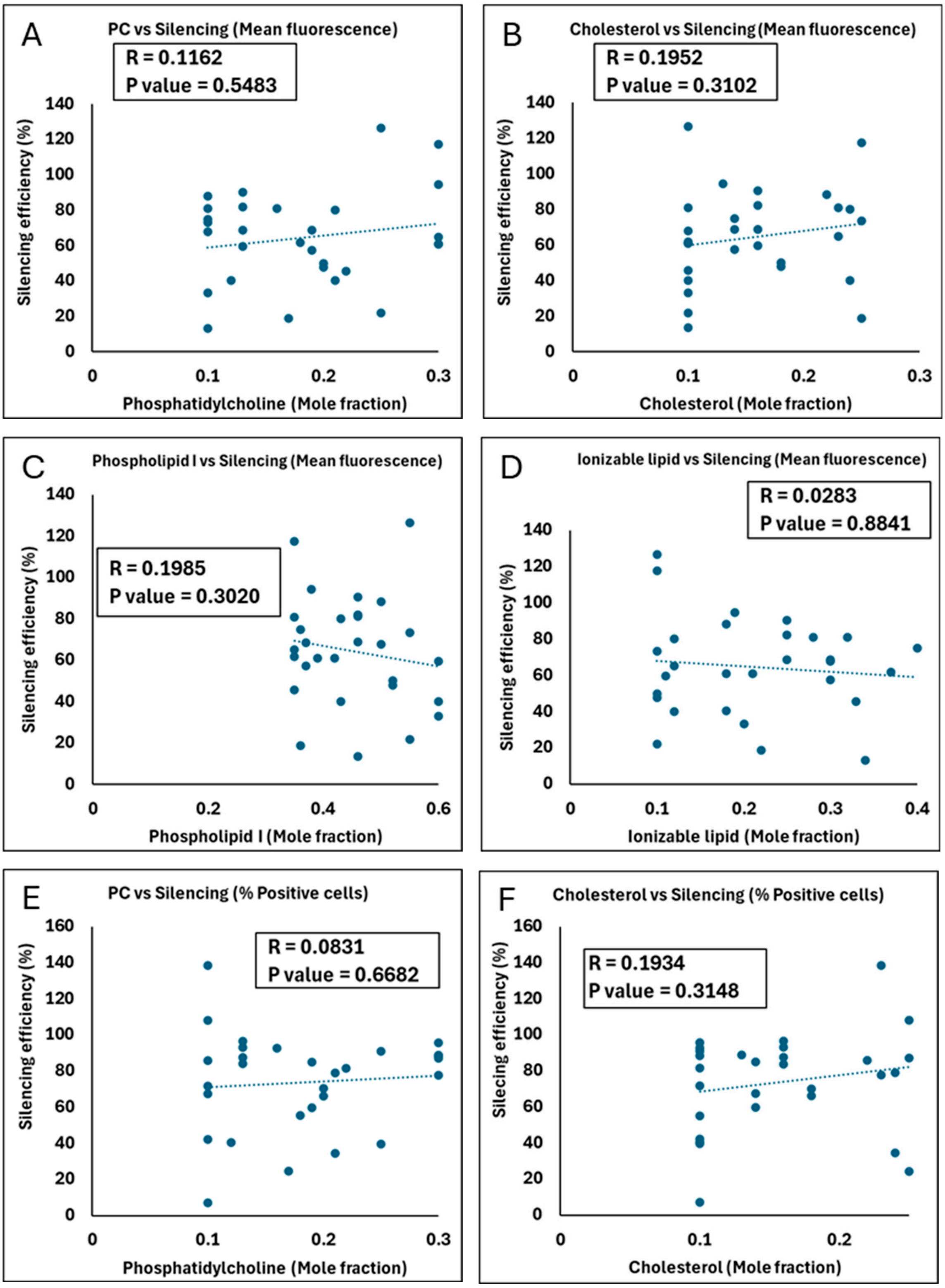
A
PC vs Silencing (Mean fluorescence)
R = 0.1162
P value = 0.5483
Silencing efficiency (%)
Phosphatidylcholine (Mole fraction)
B
Cholesterol vs Silencing (Mean fluorescence)
R = 0.1952
P value = 0.3102
Silencing efficiency (%)
Cholesterol (Mole fraction)
C
Phospholipid I vs Silencing (Mean fluorescence)
R = 0.1985
P value = 0.3020
Silencing efficiency (%)
Phospholipid I (Mole fraction)
D
Ionizable lipid vs Silencing (Mean fluorescence)
R = 0.0283
P value = 0.8841
Silencing efficiency (%)
Ionizable lipid (Mole fraction)
E
PC vs Silencing (% Positive cells)
R = 0.0831
P value = 0.6682
Silencing efficiency (%)
Phosphatidylcholine (Mole fraction)
F
Cholesterol vs Silencing (% Positive cells)
R = 0.1934
P value = 0.3148
Silecing efficiency (%)
Cholesterol (Mole fraction)

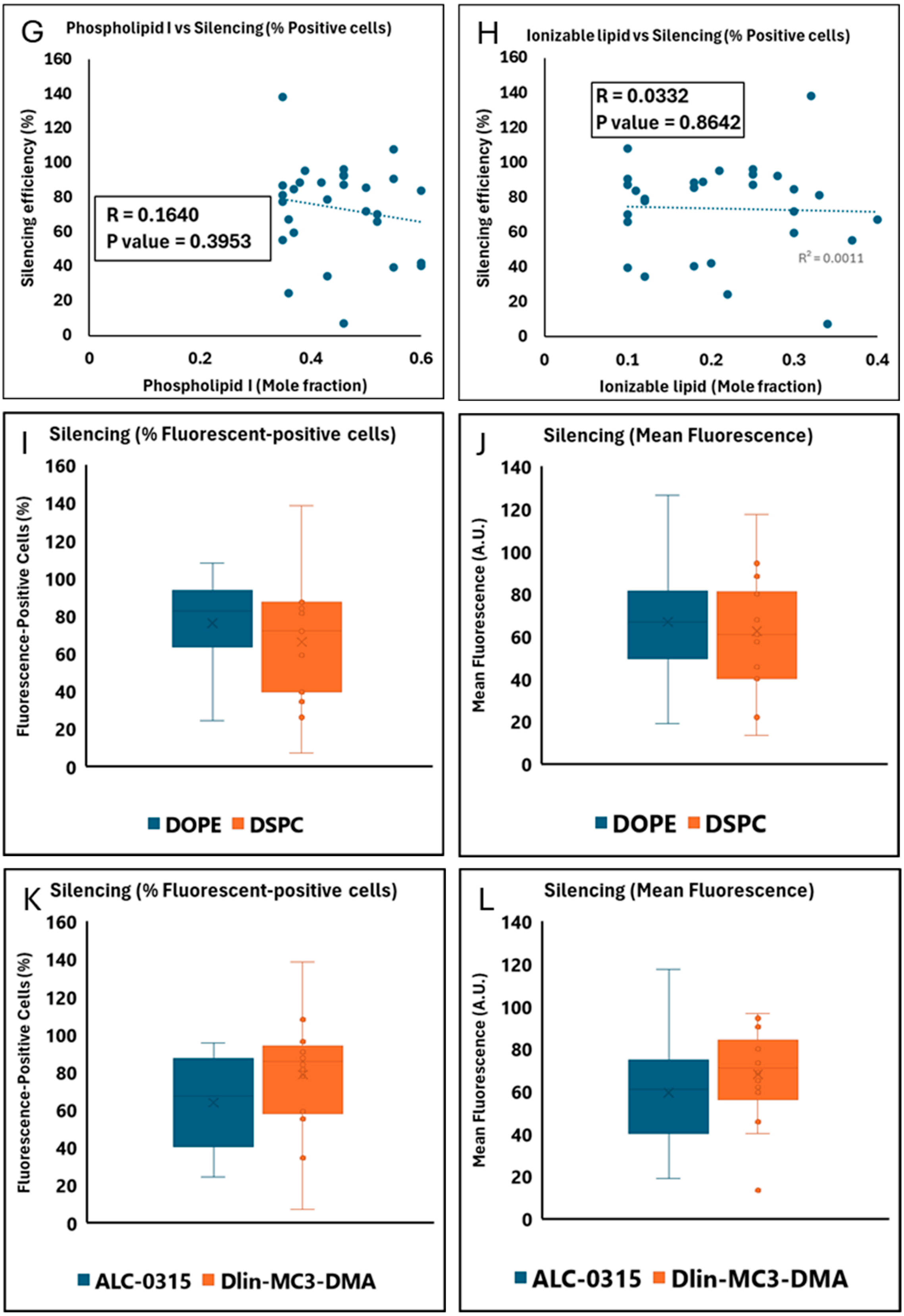
G
Phospholipid I vs Silencing (% Positive cells)
R = 0.1640
P value = 0.3953
Silencing efficiency (%)
Phospholipid I (Mole fraction)
H
Ionizable lipid vs Silencing (% Positive cells)
R = 0.0332
P value = 0.8642
R² = 0.0011
Silencing efficiency (%)
Ionizable lipid (Mole fraction)
I
Silencing (% Fluorescent-positive cells)
Fluorescence-Positive Cells (%)
DOPE DSPC
J
Silencing (Mean Fluorescence)
Mean Fluorescence (A.U.)
DOPE DSPC
K
Silencing (% Fluorescent-positive cells)
Fluorescence-Positive Cells (%)
ALC-0315 Dlin-MC3-DMA
L
Silencing (Mean Fluorescence)
Mean Fluorescence (A.U.)
ALC-0315 Dlin-MC3-DMA

Supplementary Figure S5. Investigating the effect of individual CPPs on the silencing efficiency against green fluorescent protein (GFP) in MD-MB-231 cells: Correlation between the silencing efficiency calculated based on mean fluorescence in the cell population with mole fraction of phosphatidylcholine (PC; **A**), cholesterol (**B**), Phospholipid I (**C**), and Ionizable lipid (**D**); Correlation between silencing efficiency calculated based on percentage of fluorescence-positive cells with mole fraction of phosphatidylcholine (PC; **E**), cholesterol (**F**), Phospholipid I (**G**), and Ionizable lipid (**H**); direct comparison of silencing efficiency calculated based on percentage of fluorescence-positive cells for DOPE vs DSPC (**I**) and ALC-0315 vs Dlin-MC3-DMA (**K**), and calculated based on mean fluorescence for DOPE vs DSPC (**J**) and ALC-0315 vs Dlin-MC3-DMA (**L**). No significant correlation was observed for any of the mole fractions and the indicators of the silencing efficiency. There was no significant difference between the silencing efficiency calculated based on percentage of fluorescence-positive cells (P value = 0.348) or mean fluorescence (P value = 0.667) for runs containing DOPE vs the runs containing DSPC. There was also no significant difference between the silencing efficiency calculated based on percentage of fluorescence-positive cells (P value = 0.169) or mean fluorescence (P value = 0.347) for the runs containing Dlin-MC3-DMA vs. runs with ALC-0315; however, the average of silencing efficiency for all the runs containing Dlin-MC3-DMA was higher than the average for the runs containing ALC-0315 (78.8% vs. 63.8% based on percentage of fluorescence-positive cells and 68.0% vs. 59.3% based on mean fluorescence).

Supplementary Table S3. The 28 experimental "runs" designed using the Design-Expert software to optimize the composition of Lipid/Polymer Nanoparticles (LPNPs)

Runs	DOPE	Phosphatidylcholine	Cholesterol	Dlin-MC3-DMA	Polymer	Polymer Choice
1	0.46	0.1	0.1	0.255	0.085	P5
2	0.46	0.1	0.1	0	0.34	P3
3	0.46	0.1	0.1	0	0.34	P1
4	0.46	0.1	0.1	0.255	0.085	P4
5	0.46	0.1	0.1	0.06375	0.27625	P1
6	0.46	0.1	0.1	0	0.34	P6
7	0.46	0.1	0.1	0.255	0.085	P3
8	0.46	0.1	0.1	0.1275	0.2125	P5
9	0.46	0.1	0.1	0.255	0.085	P6
10	0.46	0.1	0.1	0.1275	0.2125	P2
11	0.46	0.1	0.1	0.06375	0.27625	P3
12	0.46	0.1	0.1	0.1275	0.2125	P4
13	0.46	0.1	0.1	0.06375	0.27625	P2
14	0.46	0.1	0.1	0	0.34	P2
15	0.46	0.1	0.1	0.1275	0.2125	P6
16	0.46	0.1	0.1	0	0.34	P4
17	0.46	0.1	0.1	0.19125	0.14875	P3
18	0.46	0.1	0.1	0	0.34	P5
19	0.46	0.1	0.1	0.19125	0.14875	P2
20	0.46	0.1	0.1	0.255	0.085	P2
21	0.46	0.1	0.1	0.1275	0.2125	P3
22	0.46	0.1	0.1	0.255	0.085	P1
23	0.46	0.1	0.1	0.1275	0.2125	P1

Supplementary Table S4. The statistical analysis data for the 23 LPNP runs

Response	F-value	p-value	Sum of Squares (SS)	Mean Square (MS)	Adjusted R^2	Predicted R^2	Adequate Precision	Lack of Fit
Uptake (% fluorescence-positive cells)	7.18	0.0016	4477.73	263.4	0.7956	N/A	9.67	0.0779
Uptake (Mean Fluorescence)	37.02	<0.0001	0.0029	0.0002	0.9578	N/A	24.73	0.1167
LC50 (based on nM of siRNA delivered)	5.97	0.0034	34792.8	11597.6	0.3557	0.2383	6.32	0.0006
Silencing (based on % GFP-positive cells)	3.41	0.0266	10402.2	611.89	0.6027	N/A	6.18	0.0004
Silencing (based on Mean fluorescence)	74.78	<0.0001	10458.6	522.93	0.9820	N/A	28.7764	0.4307

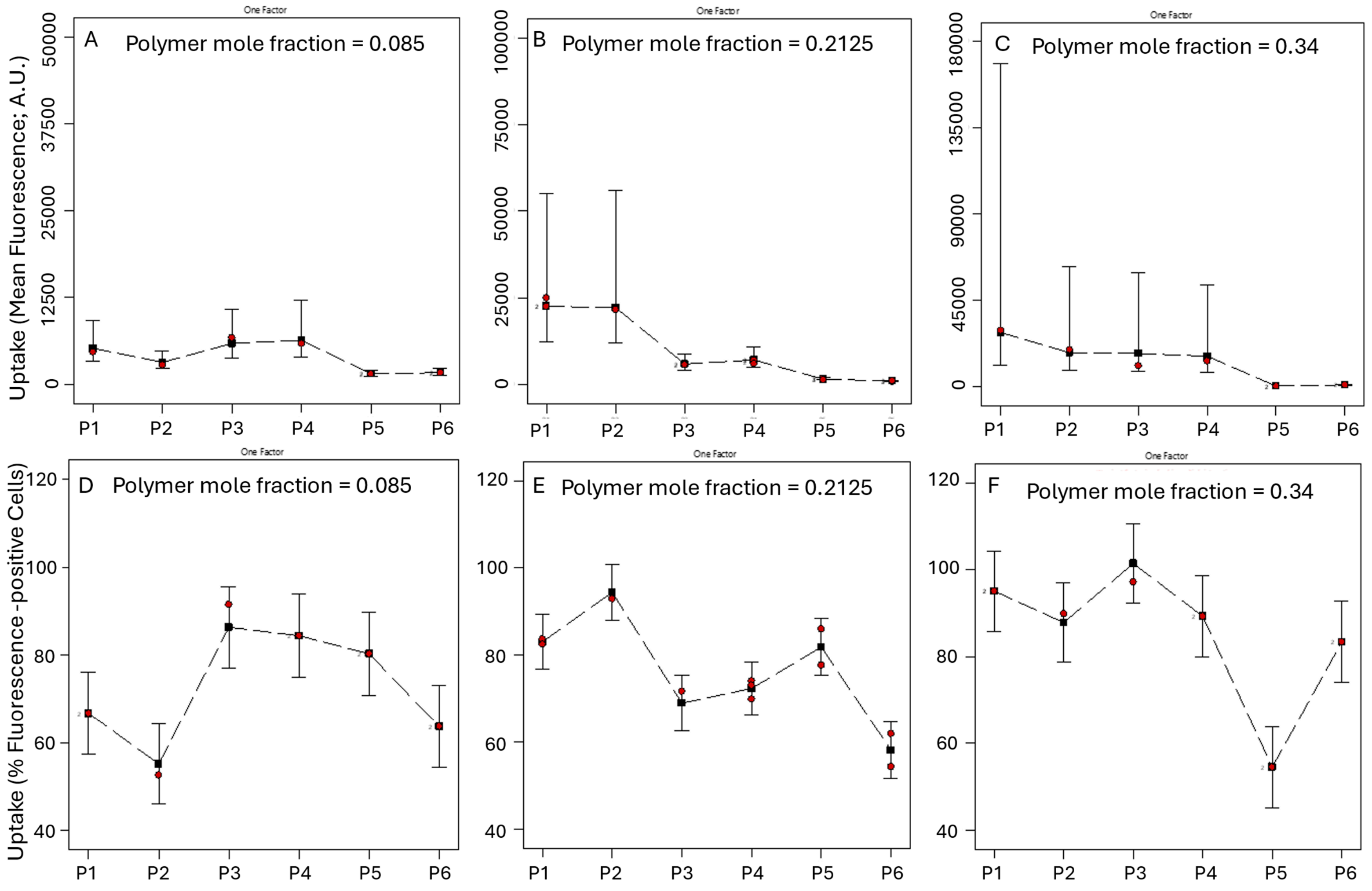

Supplementary Figure S6. The one factor respond surface plots for cellular internalization based on mean fluorescence (A.U.) for polymer mole fractions of 0.085 (**A**), 0.2125 (**B**), and 0.34 (**C**), and based on percentage of fluorescence-positive cells for polymer mole fractions of 0.085 (**D**), 0.2125 (**E**), and 0.34 (**F**).

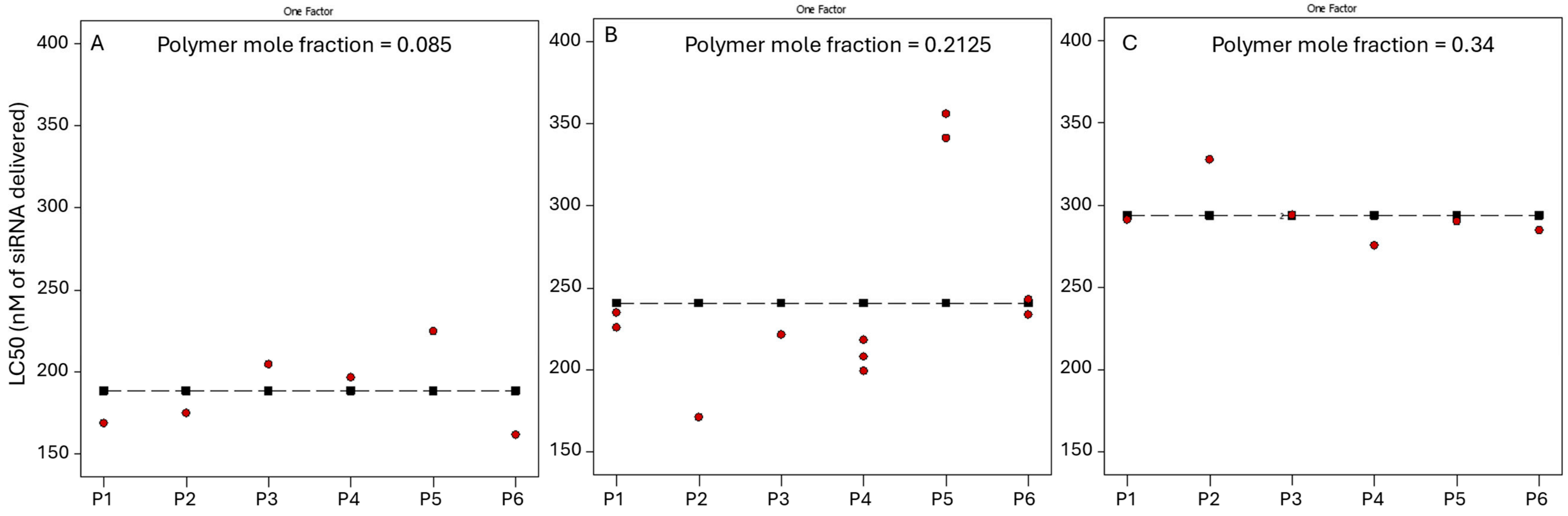

Supplementary Figure S7. The one factor respond surface plots for cytotoxicity of the LPNPs based on calculated LC50 (nM of siRNA delivered) for polymer mole fractions of 0.085 (**A**), 0.2125 (**B**), and 0.34 (**C**).

A

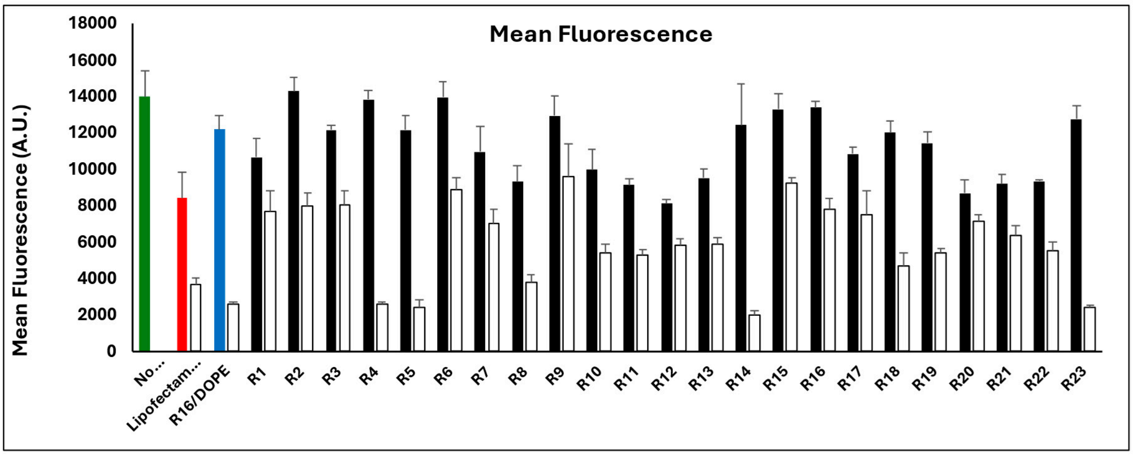

B

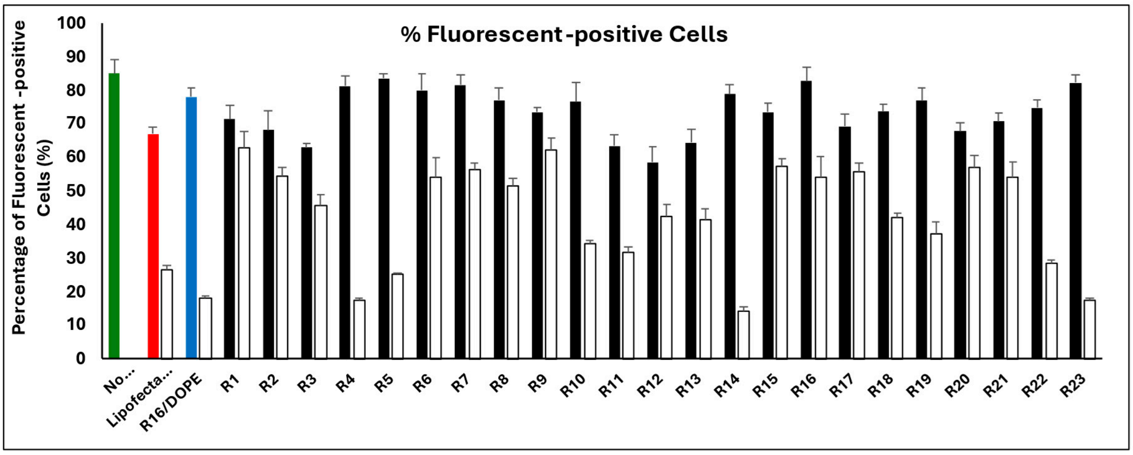

Supplementary Figure S8. The mean fluorescence (**A**) and percentage of cells positive for GFP fluorescence (**B**) for MDA-231-GFP cells exposed to scrambled or GFP targeting siRNA (100 nM final concentration) delivered by the 23 runs designed for LPNPs.

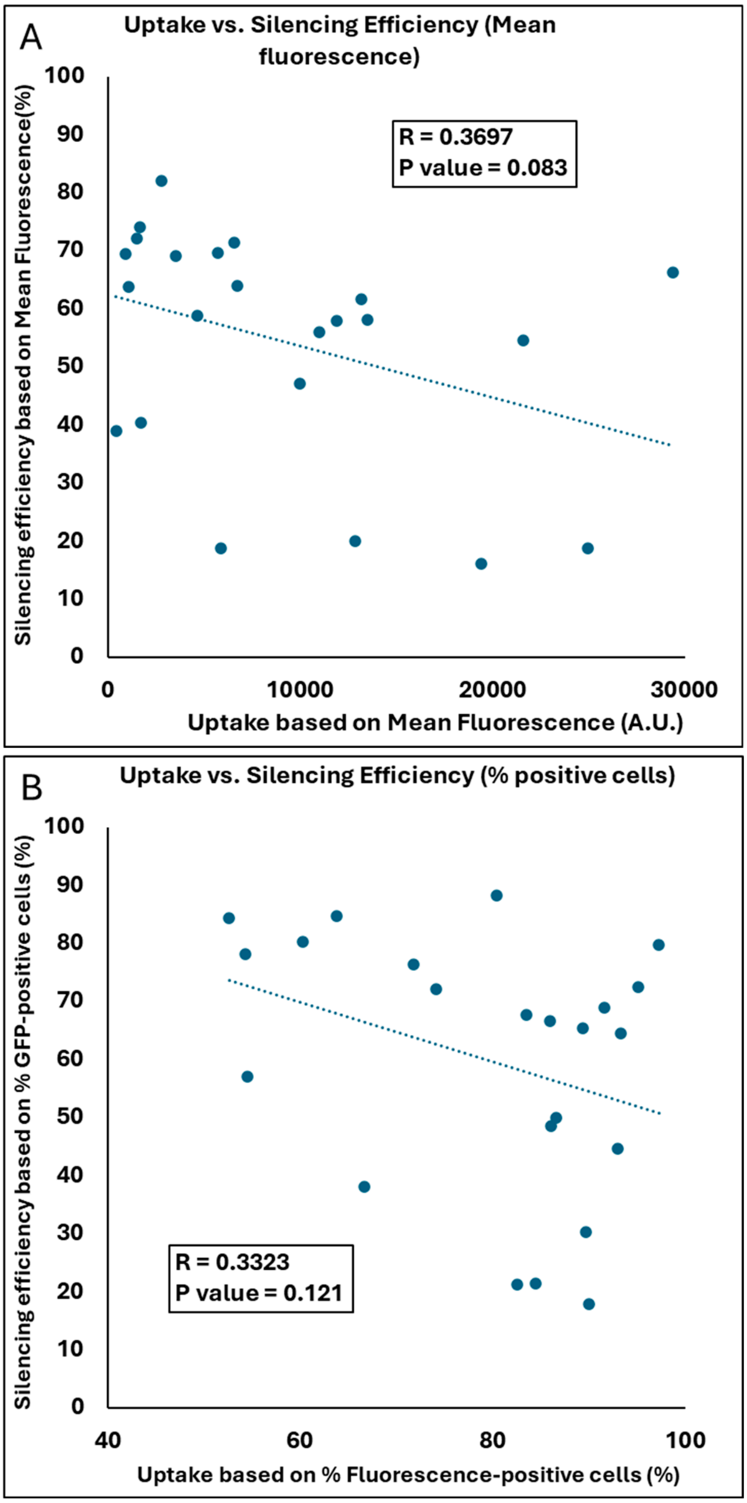

Supplementary Figure S9. Correlation between the uptake and silencing efficiency of the designed LPNP 23 runs in MDA-MB-231 and MDA-MB-GFP cells, respectively, as indicated by mean fluorescence (**A**) and percentage of fluorescence positive cells (**B**).

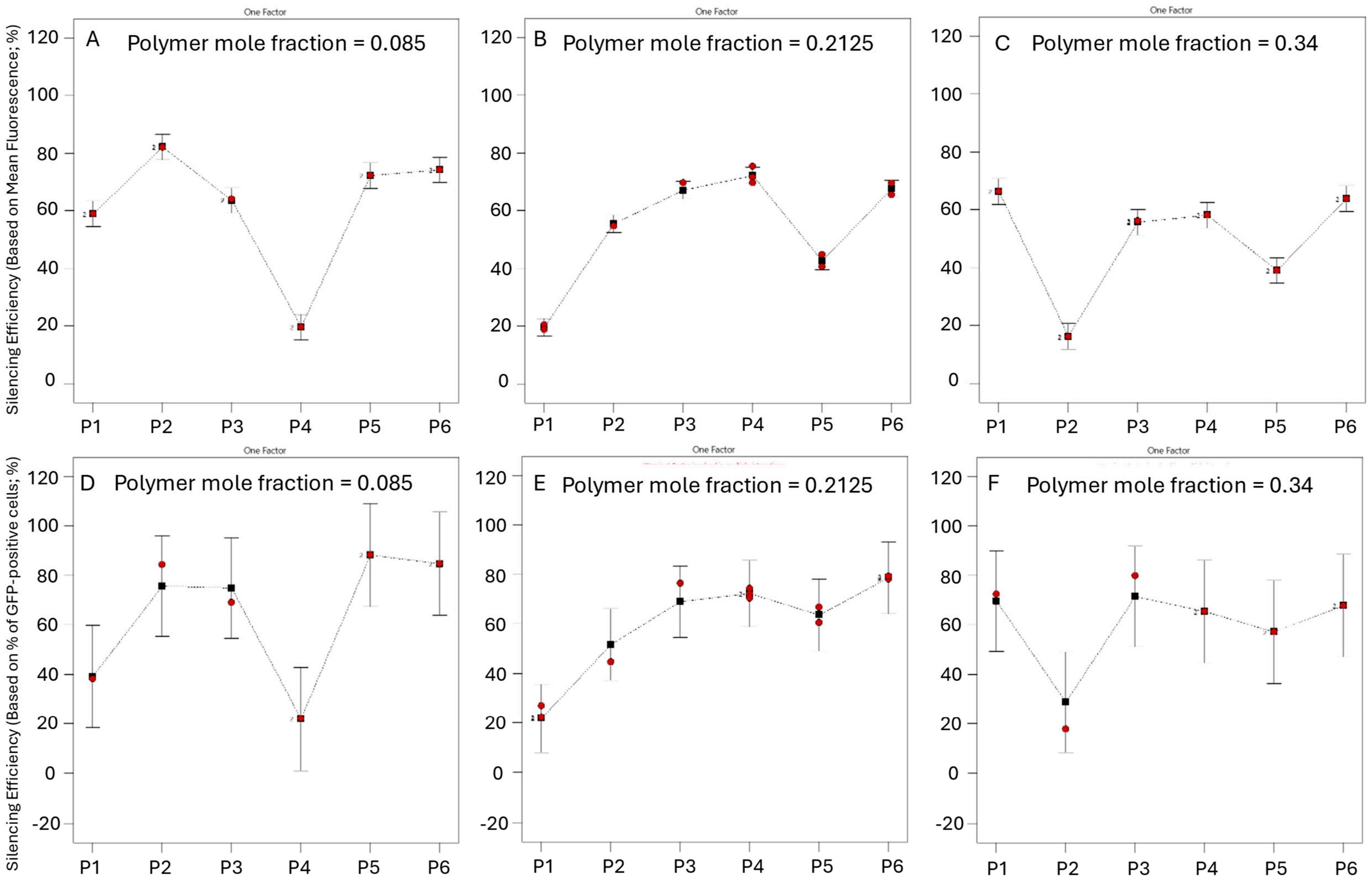

Supplementary Figure S10. The one factor respond surface plots for silencing efficiency calculated based on mean fluorescence (A.U.) for polymer mole fractions of 0.085 (**A**), 0.2125 (**B**), and 0.34 (**C**), and based on percentage of fluorescence-positive cells for polymer mole fractions of 0.085 (**D**), 0.2125 (**E**), and 0.34 (**F**).

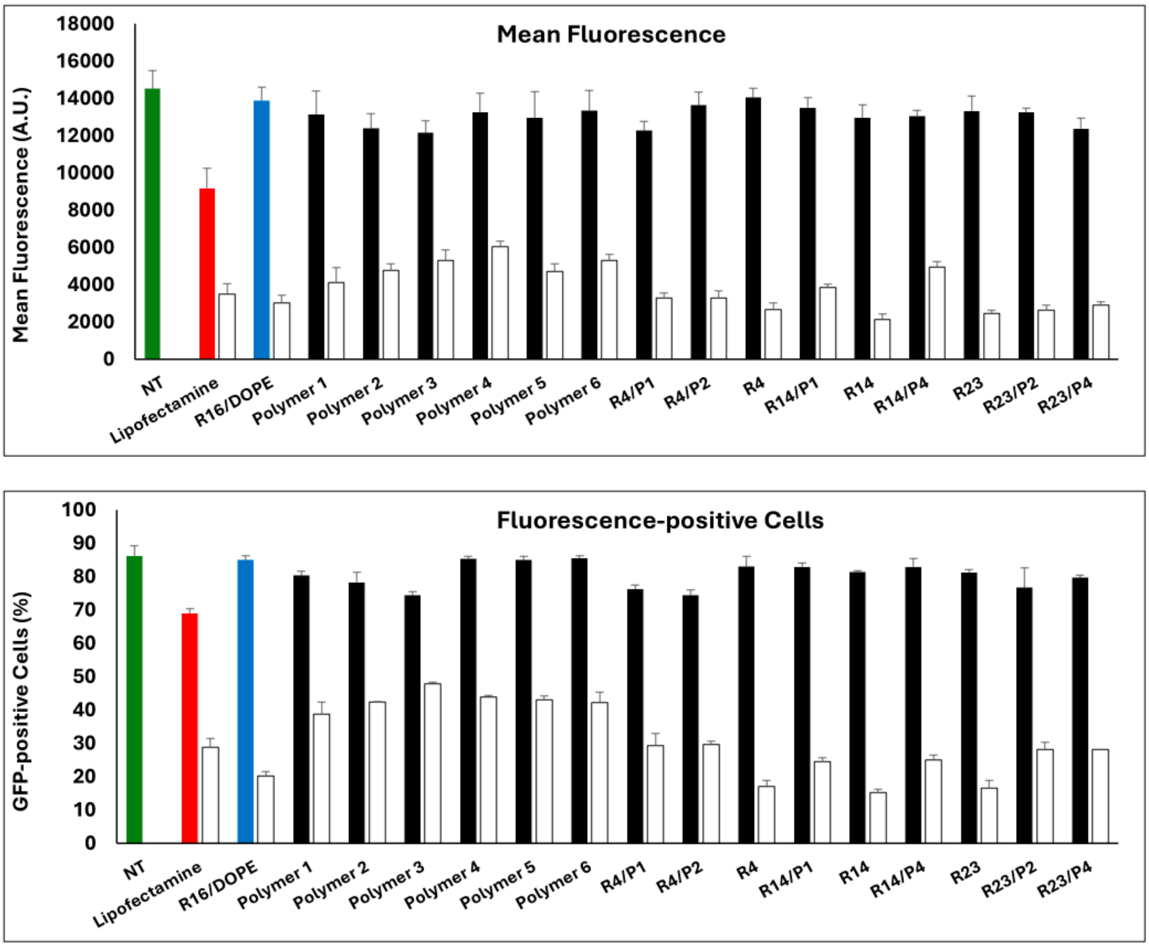

Supplementary Figure S11. The mean fluorescence (**A**) and percentage of cells positive for GFP fluorescence (**B**) for MDA-231-GFP cells exposed to scrambled or GFP targeting siRNA (100 nM final concentration) delivered by the study groups included in the experiments designed to validate the Design Expert analysis of the LPNP performance (representing the data used to calculate the silencing efficiencies presented in **Figures 10 C** and **10D**.

Supplementary Table S5. Hydrodynamic diameter, polydispersity (PD), and ζ-potential of selected LNP (Optimal formulation or OF and R16/DOPE) and LPNP formulations (R4 incorporating polymer 4 with mole fraction of 0.085, R14 incorporating polymer 2 with mole fraction of 0.34, and polymer 23 incorporating polymer 1 with mole fraction of 0.2125)

Nanoparticle	Size (nm)	PD	ζ-Potential (mV)
Optimal Formulation (OF)	103.37	0.062	-5.6 ± 2.07
R16/DOPE	99.3	0.087	-4.3 ± 2.6
Run 4 (LPNP)	101.8	0.084	8.27 ± 1.11
Run 14 (LPNP)	102	0.12	44.9 ± 5.59
Run 23 (LPNP)	101.4	0.053	38.1 ± 2.92
